# Molecular basis for cooperative binding and synergy of ATP-site and allosteric EGFR inhibitors

**DOI:** 10.1038/s41467-022-30258-y

**Published:** 2022-05-09

**Authors:** Tyler S. Beyett, Ciric To, David E. Heppner, Jaimin K. Rana, Anna M. Schmoker, Jaebong Jang, Dries J. H. De Clercq, Gabriel Gomez, David A. Scott, Nathanael S. Gray, Pasi A. Jänne, Michael J. Eck

**Affiliations:** 1 Department of Cancer Biology, Dana-Farber Cancer Institute, Boston, MA 02215 USA; 2 Department of Biological Chemistry and Molecular Pharmacology, Harvard Medical School, Boston, MA 02115 USA; 3 Lowe Center for Thoracic Oncology, Dana-Farber Cancer Institute, Boston, MA 02215 USA; 4 Department of Medical Oncology, Dana-Farber Cancer Institute, Boston, MA 02215 USA; 5 Department of Medicine, Harvard Medical School, Boston, MA 02115 USA; 6 Present Address: Department of Chemistry, University at Buffalo, Buffalo, NY 14260 USA; 7 Present Address: College of Pharmacy, Korea University, Seoul, Korea; 8 Present Address: CISTIM, Leuven, Belgium; 9 Present Address: Department of Chemical and Systems Biology, CHeM-H, Stanford Cancer Institute, Stanford University, Stanford, CA 94305 USA

**Keywords:** Kinases, X-ray crystallography, Non-small-cell lung cancer, Pharmacology

## Abstract

Lung cancer is frequently caused by activating mutations in the epidermal growth factor receptor (EGFR). Allosteric EGFR inhibitors offer promise as the next generation of therapeutics, as they are unaffected by common ATP-site resistance mutations and synergize with the drug osimertinib. Here, we examine combinations of ATP-competitive and allosteric inhibitors to better understand the molecular basis for synergy. We identify a subset of irreversible EGFR inhibitors that display positive binding cooperativity and synergy with the allosteric inhibitor JBJ-04-125-02 in several EGFR variants. Structural analysis of these complexes reveals conformational changes occur mainly in the phosphate-binding loop (P-loop). Mutation of F723 in the P-loop reduces cooperative binding and synergy, supporting a mechanism in which F723-mediated contacts between the P-loop and the allosteric inhibitor are critical for synergy. These structural and mechanistic insights will aid in the identification and development of additional inhibitor combinations with potential clinical value.

## Abstract (web summary)

Acquired drug resistance is common during chemotherapy. Here, the authors describe the structural basis and molecular mechanism by which allosteric and clinically approved, ATP-competitive inhibitors of EGFR synergize to overcome resistance in lung cancer.

## Introduction

Based on the latest global statistics, lung cancer is the most common cancer in males and has the second highest incidence cancer in females after breast cancer. Importantly, lung cancer is the leading cause of cancer-related death worldwide for both men and women^1^. While smoking is often cited as the major environmental risk for lung cancer, the incidence of lung cancer in never-smoking individuals has been on the rise. In that population, activating mutations in the epidermal growth factor receptor (EGFR), a receptor tyrosine kinase, are a major cause of non-small cell lung cancer (NSCLC)^2–4^. The most common point mutation driver of NSCLC is EGFR(L858R), which activates the kinase while increasing K_m,ATP_^5,6^. The first-generation reversible, ATP-competitive, small molecule tyrosine kinase inhibitors (TKIs) gefitinib and erlotinib selectively inhibit the L858R variant by exploiting the decreased ATP affinity of this mutant EGFR^7,8^. While most patients initially respond to treatment with reversible inhibitors, in many cases cancers become resistant through acquisition of the secondary T790M mutation. The T790M mutation occurs at the so-called gatekeeper residue and results in enhanced affinity for ATP thereby greatly decreasing the potency of reversible ATP-competitive inhibitors^9^. Second-generation irreversible inhibitors such as afatinib can inhibit the T790M variant, but are also very potent inhibitors of the WT receptor, leading to a very narrow therapeutic index and toxicity^10,11^. The emergence of the T790M mutation inspired efforts to develop agents that act in a mutant-selective manner to overcome drug resistance. The resulting third-generation inhibitors, including the proof-of-concept tool compound WZ4002 and the clinical agent osimertinib, react covalently with C797 in EGFR to overcome increased ATP affinity in the L858R/T790M variant^12–14^. However, treatment with osimertinib results in the emergence of a drug resistant variant that features the C797S mutation, which prevents formation of the covalent adduct^15^. Although the remarkable success of osimertinib has elevated it to a front-line therapy, even for patients without the secondary T790M mutation^16^, the L858R/T790M/C797S variant arises in >25% of patients over the course of treatment and is not effectively treated by currently approved EGFR TKIs^17–19^.

One promising strategy for combating these acquired resistance mutations is the development of mutant-selective allosteric EGFR inhibitors^20,21^. In contrast to ATP-competitive inhibitors, these bind in a pocket adjacent to the ATP site, not the ATP binding site itself, and inhibit the kinase by stabilizing its inactive “C-helix out” conformation. The limitation of the early allosteric inhibitors (EAI001, EAI045, and DDC4002) was their inability to inhibit EGFR in cells when applied as single agents. This behavior is a result of EGFR dimerization, which occludes the allosteric pocket by inducing the active “C-helix in” conformation. Combination treatment with allosteric inhibitor EAI045 and the dimerization-disrupting antibody cetuximab is effective against L858R/T790M and L858R/T790M/C797S models of NSCLC. Further development of allosteric EGFR TKIs has recently yielded more potent analogs of EAI045, JBJ-04-125-02 (referred to hereafter as JBJ-125) and JBJ-09-063 (referred to hereafter as JBJ-063) that are active as a single agent against L858R/T790M and L858R/T790M/C797S, likely due to their higher affinity^22,23^.

Given that ATP-competitive and allosteric inhibitors occupy different binding pockets, using them in combination may offer additional efficacy and potentially thwart resistance due to further mutations in the receptor itself. Recently, an example of dual targeting of ATP site and allosteric inhibitors against the same kinase was reported for BCR-ABL^24–26^. Unlike BCR-ABL, in which the two inhibitor binding pockets (the ATP binding site and the myristoyl site) are on distant regions of the protein, the ATP and allosteric inhibitor sites in EGFR are adjacent to one another thus raising the possibility of direct intermolecular interactions between compounds when bound within these sites. We reported that osimertinib and JBJ-125 synergize in vivo and hinder the emergence of acquired drug resistance, suggesting that combination treatment with ATP-competitive and allosteric TKIs could be viable^22^. More recently, we expanded upon this with an improved allosteric inhibitor, JBJ-063, and showed its effectiveness as a single-agent and combination therapy in a variety of TKI-resistant xenograft models^23^. In contrast, osimertinib and EAI045 do not synergize, despite the fact that they co-bind^22,27^, and WZ4002 and EAI045 appear not to co-bind due to a direct steric clash.

Here, we provide a molecular view of the mechanism by which ATP-competitive and allosteric EGFR inhibitors cooperatively bind and synergize, as obtained through a combination of structural, biochemical, and biophysical analyses. Given the adjacency of the ATP and allosteric sites and our and others’ previous finding that ATP-site and allosteric inhibitors can bind the same kinase simultaneously, it is reasonable to expect that particular pairs of ligands could do so in a manner that exhibited positive, negative, or no apparent cooperativity, and thus account for observed drug synergy in vivo. We discovered that different combinations of ATP-competitive and allosteric inhibitors can display either positive or negative cooperativity. Crystal structures of several simultaneously bound allosteric and ATP site inhibitor combinations revealed conformational changes, most notably in the phosphate-binding loop (P-loop), that may contribute to productive variations in cooperative binding. We present evidence that cooperative binding and inhibition synergy is at least partially the result of π-stacking interactions between the phenyl ring of F723 located in the P-loop and a phenyl ring in the allosteric inhibitor. This study advances our mechanistic understanding of how allosteric and ATP-competitive EGFR inhibitors synergize to target EGFR-driven cancers and provides insight to guide the future development of combination therapies.

## Results

### Crystal structures with osimertinib and allosteric inhibitors

**Fig. 1:**
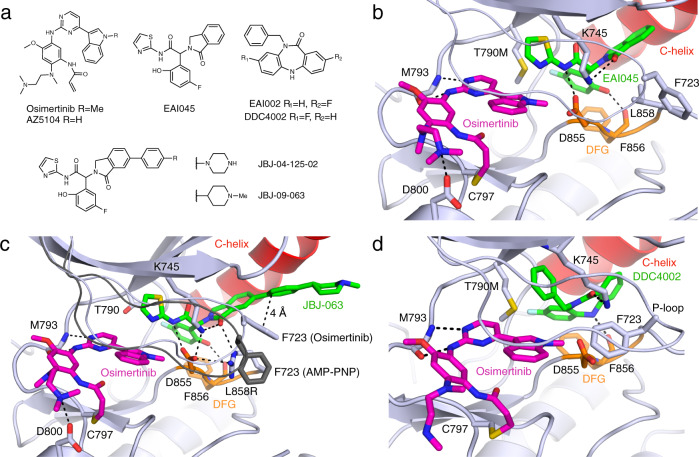
Structural characterization of osimertinib co-binding with different allosteric inhibitors. **a** Chemical structures of ATP-competitive and allosteric inhibitors. **b** Crystal structure of EGFR(T790M/V948R) in complex with osimertinib and EAI045 (PDB 7JXM). The C-helix is colored red, Asp-Phe-Gly (DFG) motif in orange, ATP-site inhibitor in magenta, and allosteric inhibitor in green. **c** EGFR(L858R/V948R) in complex with osimertinib and JBJ-063 (PDB 7K1H). The P-loop and side chain of F723 from EGFR(L858R/V948R) in complex with AMP-PNP and JBJ-063 is shown in dark gray for comparison (PDB 7K1I). **d** EGFR(T790M/V948R) in complex with osimertinib and DDC4002 (PDB 6XL4).

To establish a structural foundation for exploring co-binding of osimertinib and allosteric inhibitors, we determined EGFR(T790M/V948R) structures with a set of allosteric inhibitors and the TKI osimertinib (Fig. 1a, Supplementary Table 1, Supplementary Figs. 1–4). The V948R mutation in the kinase C-lobe aids in crystallization of the inactive conformation of the kinase, which is required for allosteric inhibitor binding. The structure of osimertinib+EAI045 closely resembles a recently reported combination structure as well as the structure of EGFR in complex with AMP−PNP+EAI045 (Fig. 1b)^21,27,28^. EAI045 belongs to the phenylglycine chemotype of allosteric EGFR inhibitors and has a tri-blade structure that binds in a pocket formed when the kinase is in the inactive conformation with the C-helix positioned outward. The carbonyl of the isoindolinone hydrogen bonds with K745, and the amide interacts with D855 in the Asp-Phe-Gly (DFG) motif. The phenol hydrogen bonds with the backbone of F856 and π-stacks with the phenyl side chain. The thiazole ring packs against T790M and engages in a S–π interaction. This binding mode does not displace ATP (Supplementary Fig. 2a).

We next determined crystal structures with osimertinib and the allosteric inhibitor JBJ-125, which unlike EAI045 synergizes with osimertinib in cells^22^. The P-loop dramatically folds downward and inwards toward the allosteric inhibitor when in complex with osimertinib and JBJ-125 or the close analog JBJ-063 in EGFR(T790M/V948R), with the side chain of F723 in the turn of the P-loop positioned within 4 Å of the phenyl ring of the allosteric inhibitor (Supplementary Fig. 2b, c). This distance and arrangement are typical of offset or T-shape π-stacking interactions^29^ and are not observed in our osimertinib+EAI045 structure, likely as a result of the lack of phenyl ring for F723 to contact. Co-binding between osimertinib or AMP-PNP and JBJ-063 was also visualized in EGFR(L858R/V948R), in which L858R increases accessibility of the allosteric pocket by disordering the inhibitory turn leading into the activation loop, which may contribute to the L858R selectivity of allosteric inhibitors (Fig. 1c, Supplementary Fig. 2d). The side chain of R858 forms a cation-π interaction with the side chain of F723, likely further stabilizing this P-loop conformation and π-stacking interaction with the allosteric inhibitor. Thus, it appears that the phenyl ring in the extended arm of JBJ-125 and JBJ-063 allows for the formation of a favorable stacking interaction with F723 when co-bound with osimertinib.

To explore co-binding modes of different allosteric inhibitor chemotypes, we determined the structure of EGFR(T790M/V948R) in complex with osimertinib and the dibenzodiazepinone allosteric inhibitor DDC4002 (Fig. 1d), which targets the same allosteric pocket as EAI045^21^. The structure confirmed the ability of these ligands to co-bind and revealed that the P-loop assumes a distinct conformation compared to structures with AMP−PNP+DDC4002 or osimertinib+EAI045. Here, the side chain of F723 was observed to fold underneath the P-loop and contact the indole of osimertinib, which resembles the conformation observed in wild-type EGFR in complex with osimertinib^28,30^. Thus, the P-loop adopts alternative orientations in osimertinib-bound crystal structures depending on the chemotype of the allosteric inhibitor.

### Identification and characterization of novel inhibitor combinations

**Fig. 2:**
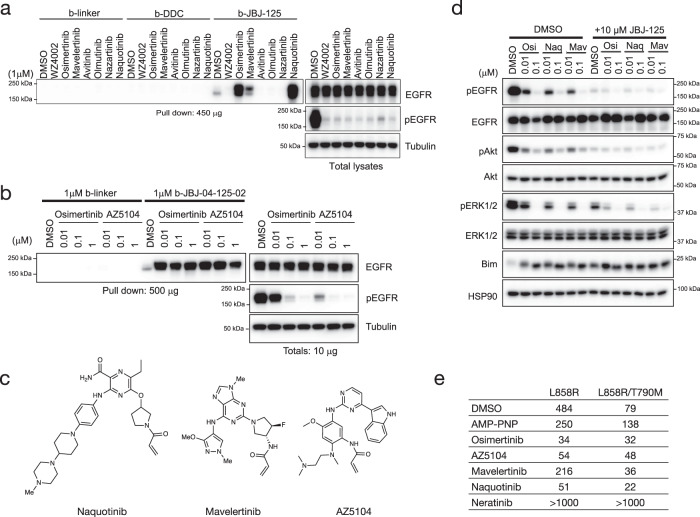
Evaluation of cooperative binding. **a** Pulldown of EGFR protein from L858R/T790M/F723A Ba/F3 cells using biotinylated allosteric inhibitor following treatment with different irreversible tyrosine kinase inhibitors (TKIs) (n = 3 independent experiments). The dibenzodiazepinone allosteric inhibitor (b-DDC) failed to pulldown EGFR, whereas pulldown with b-JBJ-125 was enhanced or abolished depending on TKI pre-treatment. Biotinylated linker (b-linker) was used as a control. **b** Pulldown of EGFR protein from L858R/T790M/F723A Ba/F3 using biotinylated JBJ-125 (b-JBJ-125) following treatment with AZ5104 (n = 3 independent experiments). **c** Chemical structures of mavelertinib, naquotinib, and the osimertinib metabolite AZ5104. **d** Inhibition synergy evaluation between osimertinib (OSI) and mavelertinib (Mav) or naquotinib (Naq) in H3255GR cells (*n* = 3 independent experiments with 3 technical replicates per experiment). **e** Fluorescence polarization (FP) binding experiments using BODIPY-JBJ and purified EGFR kinase domain. Osimertinib, AZ5104, mavelertinib, and naquotinib enhanced binding of the allosteric inhibitor. The TKI neratinib, which extends into the allosteric pocket, was prevented binding of the allosteric inhibitor. Reported as mean (*n* = 2 independent experiments). Source data are provided as a Source Data file.

To better understand the chemical diversity of third-generation ATP-site inhibitors that co-bind with JBJ-125, we employed a pulldown assay using a biotinylated analog of JBJ-125 (b-JBJ-125). We previously found that osimertinib dramatically enhances the ability of this allosteric probe to precipitate EGFR(L858R/T790M) from cell lysates, whereas afatinib and WZ4002 do not, likely as the result of steric clashes or incompatible functional groups of these inhibitors^22^. Here, we expanded upon these initial findings and examined precipitation of five additional third-generation EGFR TKIs as well as AZ5104, an active metabolite of osimertinib that binds the mutant kinase in an analogous manner (Supplementary Fig. 5). As with osimertinib, precipitation of EGFR(L858R/T790M) with biotinylated JBJ-125 was enhanced upon treatment with mavelertinib, naquotinib, and AZ5104 (Fig. 2a–c)^12,13^. By contrast, avitinib, olmutinib, and nazartinib prevented precipitation of EGFR(L858R/T790M) with b-JBJ-125, likely due to steric clashes (Supplementary Fig. 6). Guided by our crystal structure of osimertinib+DDC4002 (Fig. 1d), we also prepared the biotinylated dibenzodiazepinone allosteric inhibitor b-DDC (compound DDC-03-033-01) and tested it in cells expressing EGFR(L858R/T790M) (Supplementary Fig. 7a, Supplementary Methods). This probe did not precipitate the mutant receptor with or without the addition of ATP-site inhibitors (Fig. 2a), perhaps due to the lower potency of the dibenzodiazepinone core as compared to phenylglycine inhibitors like JBJ-125^21^.

We next tested the impacts on EGFR inhibition and subsequent downstream signals with combinations of mavelertinib or naquotinib with JBJ-125 in the NSCLC cell line H3255GR, which harbors EGFR(L858R/T790M). H3255GR cells were treated with mavelertinib or naquotinib alone or in combination with 10 μM JBJ-125 (Fig. 2d). Although either inhibitor alone suppressed phospho-EGFR, combination treatment yielded more complete inhibition of EGFR downstream signaling as evidenced by profound inhibition of pAKT and pERK1/2, which have been shown to be needed in addition to decreased phospho-EGFR to induce cell death^22^. Taken together, our results indicate that mavelertinib and naquotinib, like osimertinib, can co-bind with JBJ-125 leading to enhanced inhibition of EGFR signaling in cells.

### Binding studies between TKIs and JBJ-125

Enhanced EGFR precipitation by the biotinylated allosteric probe in the presence of certain ATP-site inhibitors suggests that a bound ATP-site inhibitor may increase affinity for JBJ-125. Such an effect could underlie cellular and in vivo synergy. To directly test this possibility, we used fluorescence polarization (FP) to examine the binding of a BODIPY-labeled version of JBJ-125 (BODIPY-JBJ, compound JBJ-09-052) to the EGFR(L858R/T790M) kinase domain (Supplementary Fig. 7b, Supplementary Methods). Though slightly less potent than JBJ-125, BODIPY-JBJ inhibits EGFR(L858R/T790M) in a mutant-selective manner with a sub-nanomolar IC_50_ (Supplementary Fig. 8a). To examine effect on co-binding, EGFR kinase domain protein was preincubated with an excess of irreversible ATP-site inhibitor, DMSO, or the non-hydrolyzable ATP analog AMP-PNP prior to titration into a solution of the fluorescently-labeled allosteric inhibitor. Pre-binding with osimertinib, AZ5104, mavelertinib, or naquotinib increased the binding affinity of the allosteric inhibitor (Fig. 2e, Supplementary Fig. 8b). The increase in binding affinity ranged from approximately 4 to 6-fold, as compared with the AMP-PNP control. Interestingly, the binding of BODIPY-JBJ was modestly disfavored in the presence of the nucleotide analog as compared with DMSO. The covalent TKI neratinib, which extends into the allosteric pocket, effectively abolished binding of BODIPY-JBJ, providing further evidence that the probe binds the allosteric pocket as expected. These results directly demonstrate that the allosteric probe binds with higher affinity when the ATP-site is occupied with naquotinib, mavelertinib, or osimertinib or its metabolite AZ5104.

### Evaluation of synergy using an enzyme inhibition assay

**Fig. 3:**
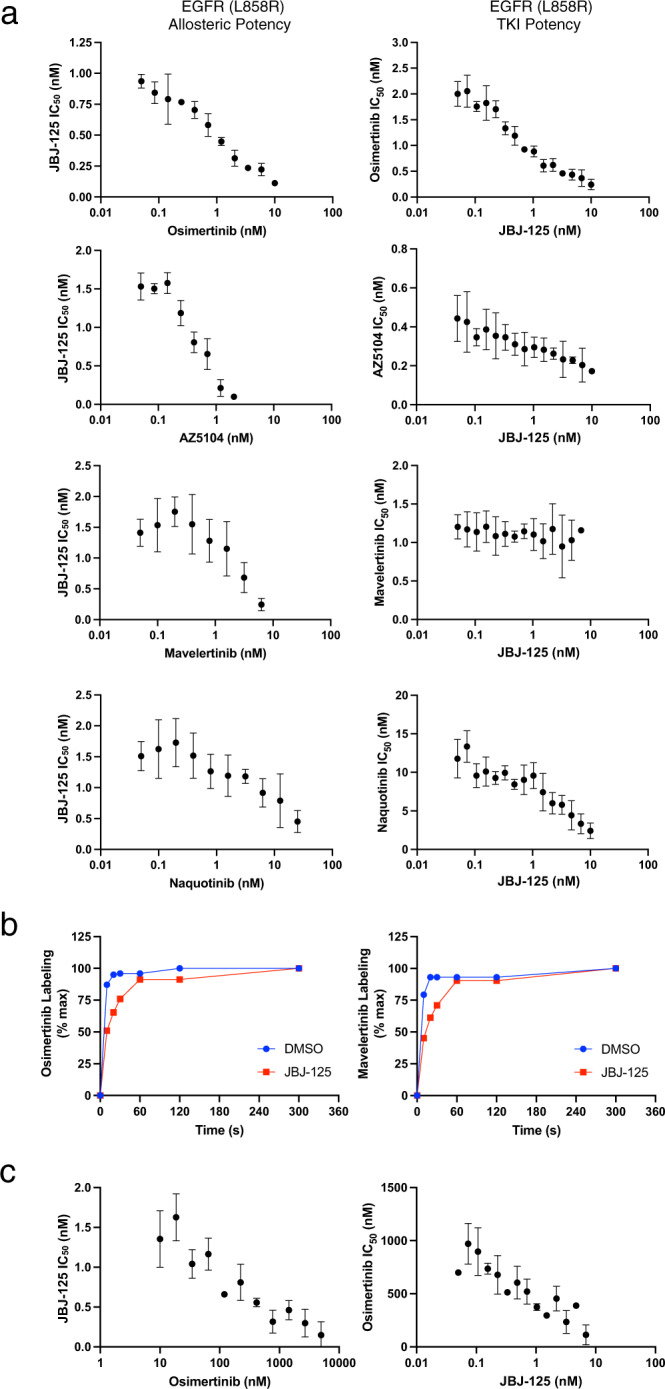
Biochemical evaluation of inhibition synergy. **a** Inhibition synergy analysis using purified EGFR kinase domain. Compound combinations were dispensed using a digital drug dispenser and IC_50_s of one inhibitor were plotted as a function of the concentration of the other inhibitor. Similar to the FP binding experiments, osimertinib, AZ5104, and naquotinib enhanced the potency of JBJ-125, and vice versa. Mavelertinib was neither synergistic nor antagonistic with regard to JBJ-125 potency. Reported as mean ± SD (*n* = 3 independent experiments). **b** Labeling by osimertinib or mavelertinib in the presence or absence of allosteric inhibitor. Percent labeling was assessed via intact mass spectrometry. Reported as mean (*n* = 2 independent experiments). **c** Synergy evaluation with EGFR(L858R/C797S) and the osimertinib+JBJ−125 combination. The allosteric inhibitor enhances the potency of osimertinib despite the C797S mutation. Reported as mean ± SD (*n* = 3 independent experiments). Source data are provided as a Source Data file.

We next examined the effects of positive or negative cooperative binding on inhibitor potencies using an in vitro kinase inhibition assay with a dose-response matrix of the two inhibitors. This approach allows determination of the IC_50_ of the allosteric inhibitor in the presence of increasing concentrations of the orthosteric inhibitor and vice versa (Supplementary Fig. 9a). Synergistic inhibitor combinations are expected to display enhanced potency in combination (lower IC_50_), while antagonistic combinations are expected to have an opposite effect (increase in IC_50_) (Supplementary Fig. 9b, c). Co-binding without synergy or antagonism should not alter the individual inhibitor potencies. We carried out this study with the L858R mutant for which osimertinib is the front-line therapy and JBJ-125, which is less potent than JBJ-063 and therefore easier to assess synergy with. With our model inhibitor combination, the presence of osimertinib decreases the IC_50_ of JBJ-125 by approximately an order of magnitude and the allosteric inhibitor has a similar effect on the potency of osimertinib (Fig. 3a). Similar synergy is observed with allosteric inhibitor JBJ-063 in combination with osimertinib (Supplementary Fig. 9d), and with the osimertinib metabolite AZ5104 in combination with JBJ-125 (Fig. 3a). By contrast, gefitinib and neratinib, which sterically overlap with the allosteric site, decrease the observed potency of the allosteric inhibitor (Supplementary Fig. 9e). In assays with wild-type EGFR, we also observed synergy between osimertinib and JBJ-125, but only at much higher inhibitor concentrations due to the mutant-selectivity of both of these agents (Supplementary Fig. 9f). Furthermore, combination of osimertinib and the dibenzodiazepinone allosteric inhibitor EAI002^21^, which is closely related to DDC4002 in our crystal structure, did not reveal synergy even at concentrations of EAI002 > 10 µM (Supplementary Fig. 9g).

ATP-site inhibitors mavelertinib and naquotinib also synergized with allosteric inhibitor JBJ-125 (Fig. 3a), but interestingly the observed synergy was not reciprocal with mavelertinib despite cooperatively binding (Fig. 2e). Mavelertinib decreased the IC_50_ of the allosteric inhibitor, but the observed potency of mavelertinib was unchanged in the presence of increasing concentrations of JBJ-125. While conservation of energy dictates that the effect of reversible binding of two ligands to the kinase must be reciprocal, these ATP-site inhibitors are irreversible^31^. Furthermore, this enzyme inhibition assay does not measure the equilibrium binding of these two ligands; it is a complex kinetic process that involves reversible and covalent binding as well as competition for binding with nucleotide. Irreversible enzyme inhibition by the orthosteric inhibitor proceeds via a reversible binding step followed by covalent bond formation, and in these inhibition assays we are measuring the net effect of both reversible and irreversible binding and inhibition. Because changes in the rate of the irreversible step could mask changes in reversible affinity, we used mass spectrometry to assess the effect of JBJ-125 on the rate of covalent bond formation of osimertinib and mavelertinib. We observed that JBJ-125 slightly slows the rate of covalent bond formation for osimertinib and mavelertinib (Fig. 3b, Supplementary Fig. 11). Thus, we suspect that the allosteric inhibitor enhances the reversible binding of mavelertinib but slows the rate of covalent bond formation such that there is the little net effect on its apparent potency in the enzyme inhibition assay.

Importantly, osimertinib and JBJ-125 also exhibited synergy with the L858R/C797S EGFR variant, which is resistant to osimertinib due to the inability to form a covalent adduct with the inhibitor. The addition of JBJ-125 enhanced the apparent potency of osimertinib by an order of magnitude (Fig. 3c). This enhancement in reversible binding explains our observations that this combination is synergistic despite slowed covalent adduct formation and is also in line with our prior results showing that osimertinib plus JBJ-125 is more effective than the allosteric inhibitor alone in osimertinib-resistant cells with the C797S mutation^22^. The compound BI-4020, a potent inhibitor of the C797S variant^32^ that is expected to sterically clash with JBJ-125, did not synergize in this assay (Supplementary Fig. 9e). Taken together, our inhibition data support the notion that synergy arises from cooperative binding of the allosteric and orthosteric inhibitors.

### Insights into structural basis for inhibitor cooperativity

**Fig. 4:**
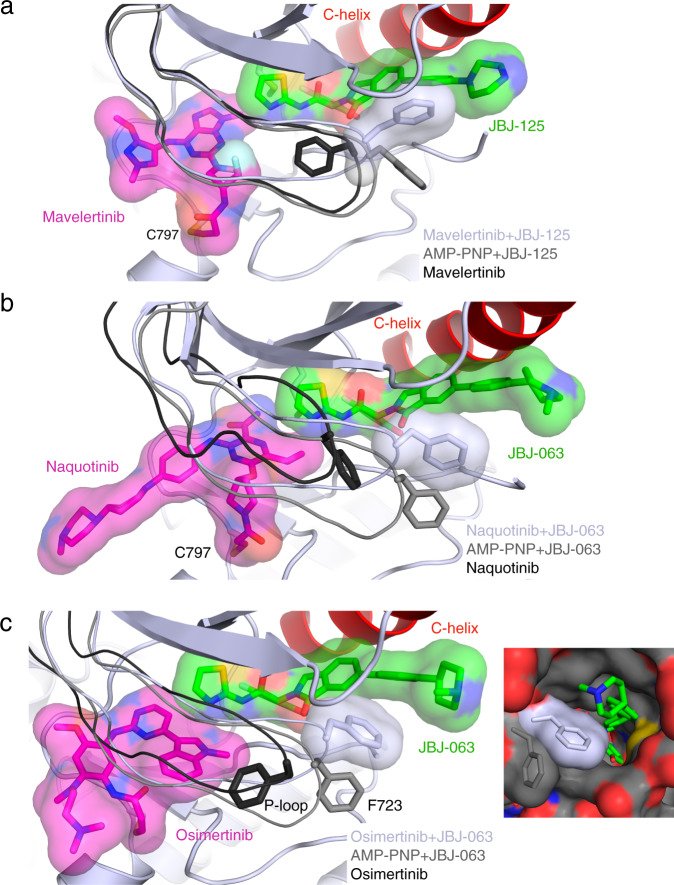
Structural characterization of additional inhibitor combinations. **a** Structure of EGFR(T790M/V948R) in complex with mavelertinib and JBJ-125 (PDB 7JXK, light gray) and P-loop comparison with AMP-PNP+JBJ-125 (PDB 7JXP, gray) and mavelertinib (PDB 7JXI, black). **b** Crystal structure of EGFR(T790M/V948R) in complex with naquotinib and JBJ-063 (PDB 7LG8, light gray) and P-loop comparison with AMP-PNP+JBJ-063 (PDB 7K1I, gray) and naquotinib (PDB 5Y9T, black). **c** Crystal structure of EGFR(L858R/V948R) in complex with osimertinib and JBJ-063 (PDB 7K1I). Comparison of P-loop conformation in complex with osimertinib (PDB 4ZAU) in black, AMP−PNP+JBJ−063 in gray (PDB 7JXQ), and osimertinib+JBJ−063 in light gray. F723 contacts the allosteric inhibitor and occludes the putative entrance and exit tunnel to the allosteric site.

Our structural analysis of mutant EGFR with osimertinib and JBJ-125 and JBJ-063 revealed rearrangement of the P-Loop as a key structural alteration that may lead to positive cooperativity through direct intermolecular π-stacking interactions with the allosteric inhibitor (Fig. 1c). To further explore the structural basis of cooperative binding of orthosteric and allosteric inhibitors, we determined co-crystal structures with mavelertinib+JBJ-125 and naquotinib+JBJ-063 (Fig. 4a, b, Supplementary Fig. 2e, f). In the structure of EGFR(T79M/V948R) and mavelertinib alone, F723 is positioned under the P-loop, but in combination with JBJ-125, the P-loop adopts a conformation similar to that of the osimertinib+JBJ-125 crystal structure (Fig. 4a) in which F723 extends to contact JBJ-125. A similar conformation is observed in the co-structure with naquotinib and JBJ-063, in which a folded P-loop conformation positions the side chain of F723 against the phenyl ring of the allosteric inhibitor (Fig. 4b). In further support of the role of this interaction in cooperativity, we find that an allosteric inhibitor that lacks the phenyl ring (EAI045) exhibits little synergy with osimertinib (Supplementary Fig. 8h). Finally, the repositioning of F723 with synergistic inhibitor pairs also constricts the exit tunnel to the allosteric site, which may enhance the affinity of JBJ-125 and JBJ-063 (Fig. 4c). Overall, our structures show that cooperative combinations of ATP-competitive and allosteric inhibitors assume the same putative cooperative conformation driven by a π-stacking interaction between F723 and the phenyl ring of the extended allosteric inhibitors (Supplementary Fig. 11), whereas other combinations either sterically clash or induce a conformation that disfavors co-binding.

**Fig. 5:**
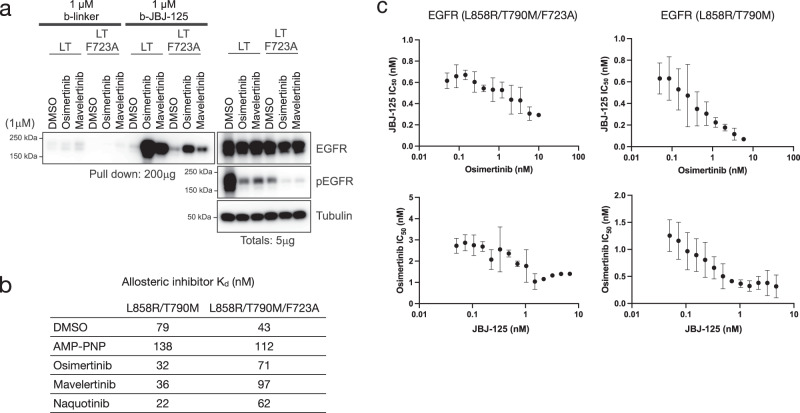
Inhibitor features and role of F723 in cooperative binding and synergy. **a** Pulldown of EGFR protein from HEK293T/17 cells transiently expressing EGFR(L858R/T790M) or EGFR(L858R/T790M/F723A) using b-JBJ-125 following treatment with different irreversible TKIs. F723A decreased the ability of select TKIs to enhance pulldown using b-JBJ-125 (*n* = 3 independent experiments). **b** Cooperative binding was not observed in the F723A variant in an FP binding assay using BODIPY-JBJ. Data are reported as mean (*n* = 2 independent experiments) EGFR(L858R/T790M) data are repeated from Fig. 2e for comparison. **c** Inhibition synergy resulted in a 20-fold increase in potency for JBJ-125 in the presence of osimertinib compared to approximately 2-fold in the L858R/T790M/F723A variant (top). Similarly, only a 3-fold increase in osimertinib potency was observed in the F723A variant compared to a 7.5-fold increase in L858R/T790M (bottom). Data are reported as mean ± SD (*n* = 3 independent experiments). Source data are provided as a Source Data file.

**Fig. 6:**
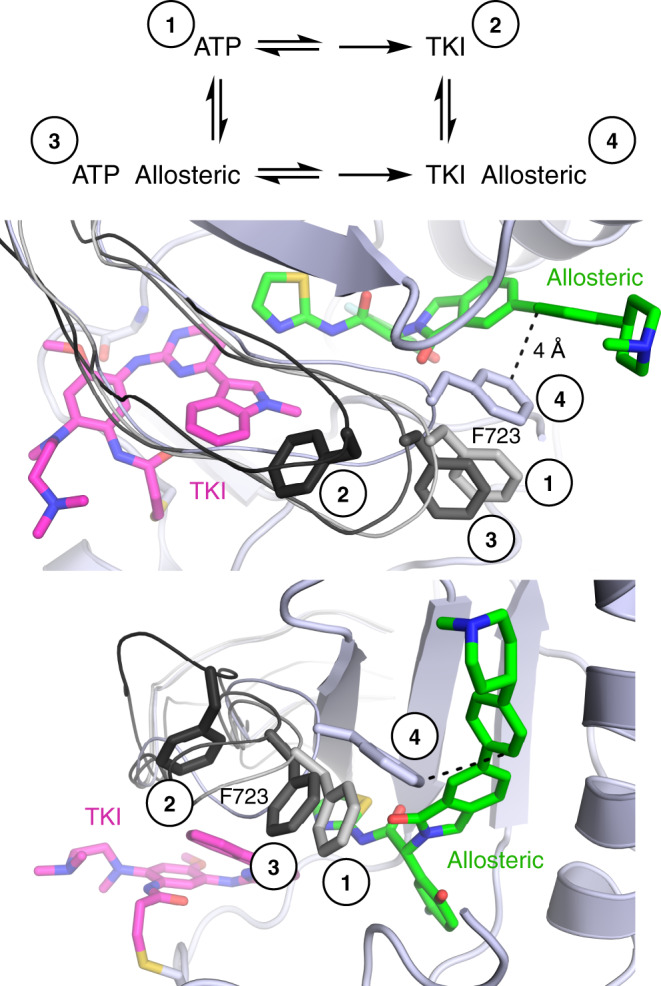
Proposed structural mechanism underlying cooperative binding and inhibition synergy. When bound to nucleotide, the phosphates force the P-loop to be in an extended conformation with the side chain of F723 outward (grays, labeled 1 and 3). When bound to a TKI (osimertinib shown, magenta), F723 often folds under the P-loop (black, labeled 2) where the phosphates of ATP would be located. When in complex with both TKI and an allosteric inhibitor (green), the P-loop folds down into the space usually occupied by the phosphates and the side chain of F723 forms a π-stacking interaction with the inhibitor (light gray, labeled 4), closing the putative exit tunnel (see Fig. 4c). This more compact ternary complex conformation facilitates cooperative binding, which leads to inhibition synergy.

To further examine the role of F723 and the P-loop rearrangement in cooperative inhibitor binding, we tested the effect of an F723A mutant on co-binding and inhibition synergy. Introduction of the F723A mutation (in the context of L858R/T790M EGFR) markedly decreased pulldown of mutant EGFR in HEK293 cells treated with osimertinib or mavelertinib (Fig. 5a). Similarly, pulldowns performed using purified EGFR(L858R/T790M/F723A) kinase domain showed a reduction in the ability of osimertinib to enhance pulldown despite the fact that the mutation did not have a major effect on potency of osimertinib and allosteric inhibitors (Supplementary Fig. 8a, 12a). Cooperative binding in the F723A variant was diminished (Fig. 5b, Supplementary Fig. 12b), and inhibition synergy was also diminished with the enhancement in JBJ-125 potency only increasing 2-fold compared to approximately 10-fold for EGFR(L858R/T790M) (Fig. 5c). Taken together, these data support a mechanism of cooperative binding of certain ATP site and allosteric inhibitors where anchoring a π-stack and hydrophobic contacts between F723 and the phenyl ring on the extended allosteric inhibitors is responsible for enhanced inhibitor binding and inhibition synergy (Fig. 6).

## Discussion

The heterogeneous nature of tumors and acquired resistance complicate therapeutic intervention for many cancers. One strategy to overcome drug resistance and achieve improved efficacy is combination therapy, where two or more targeted therapies are used simultaneously. Here, the largest therapeutic benefit can be accomplished when drugs act synergistically, i.e. when the combined effect of two drugs is beyond what can be expected if individual effects are simply added together. Synergistic drug combinations allow for the use of lower doses of each drug than in monotherapies or additive drug combinations, which may reduce adverse side effects. Additionally, synergistic drug combinations may also slow emergence of drug resistant mutants^16^, further highlighting the clinical benefit of drug combinations or polytherapy^33,34^. The vast majority of combination therapies used in oncology target multiple proteins, thus exerting enhanced effect by inhibiting concurrent oncogenic drivers or compensatory pathways. In the present study, we find that the enhanced efficacy of the ATP-site EGFR inhibitor osimertinib and allosteric inhibitor JBJ-125 when used in combination is due, at least in part, to cooperativity; enhanced affinity of one agent for the mutant receptor in the presence of the other.

Not surprisingly, positive cooperativity is dependent on the chemical structure of both the ATP-site and allosteric agents. We observe synergistic inhibition and cooperative binding with a subset of third-generation irreversible inhibitors, including mavelertinib and naquotinib in addition to osimertinib (Figs. 2, 3). Conversely, ATP-site inhibitors that are sterically incompatible with the allosteric inhibitors, such as gefitinib or neratinib, do not synergize and they antagonize binding of the allosteric inhibitor (Supplementary Fig. 9e). On the allosteric side, JBJ-125 and the related compound JBJ-063 exhibit synergy and apparent cooperative binding while DDC002 does not. EAI001 does not synergize in cellular assays^22^ and exhibits only very modest synergy in our biochemical inhibition assays. Importantly, cooperativity is only one of several factors that may contribute to synergistic activity at the cellular and in vivo level. For example, gefitinib cannot co-bind with JBJ-125 and is antagonistic in our biochemical assay but may nevertheless synergize with JBJ-125 via more potent inhibition of certain EGFR alleles that may arise due to tumor heterogeneity or in the context of EGFR amplification. The non-small cell lung cancer cell line H3255 harbors amplified EGFR(L858R), but in the gefitinib-resistant derivative cell line H3255GR only a subset up alleles carry the additional T790M mutation that confers resistance to gefitinib but sensitizes to the allosteric inhibitor^35^.

Our structural analysis implicates the P-loop conformation as a central structural feature that mediates cooperative binding of ATP-site and allosteric inhibitors. In all co-structures with cooperative inhibitor combinations, the P-loop folds downward into the space typically occupied by the phosphates of the ATP and positions the side chain of F723 against the phenyl ring in the allosteric inhibitor (Fig. 6). This rearrangement provides apparently favorable hydrophobic contacts and π-stacking between this phenylalanine residue and the allosteric inhibitor. In support of this model, mutation of F723 to alanine diminishes synergy and cooperativity as does the use of EAI045 as the allosteric inhibitor (Fig. 5, Supplementary Fig. 9h). EAI045 is similar to JBJ-125 but lacks the interacting phenyl ring (Fig. 1a, b). Since producing NSCLC cell lines with the F723A mutation has proven challenging, we overexpressed the receptor in HEK293 cells, which provides sufficient receptor with which to perform pulldown experiments. We were also unable to produce EGFR(L858R/T790M/F723A) Ba/F3 cells, likely due to reduced basal signaling (Fig. 5a). Thus, we were not able to use this mutation to assess the extent to which cooperative binding of allosteric and ATP-site inhibitors explains the synergy we observe in a cellular context.

A rare example of co-targeting a single protein is BCR-ABL, which can be co-drugged with asciminib (ABL001), a myristoyl-site allosteric inhibitor, and an ATP-site TKI such as nilotinib or ponatinib^26,36^. Unlike the allosteric site in EGFR, the ABL myristoyl pocket allosteric site is remote from kinase active site^37,38^. Simultaneous binding of nilotinib and asciminib to ABL has been confirmed by X-ray crystallography, but cellular synergy studies indicate that their antiproliferative effects are additive but not synergistic^36^. Ponatinib and asciminib appear to synergistically inhibit certain compound mutants that are resistant to either agent alone, and cooperativity has been suggested as a possible underlying mechanism^26^. However, to our knowledge biochemical cooperativity has not been demonstrated for ATP- and myristoyl-site inhibitors of BCR-ABL. Interestingly, cooperative binding of allosteric and ATP-site ligands has recently been demonstrated for a cyclin-dependent kinase. The fluorescent probe ANS can bind a similar C-helix-out allosteric site in CDK2, and its affinity is increased several fold by binding of roscovitine in the ATP-site^39^.

Although the pharmacokinetic properties of JBJ-125 and JBJ-063 limit their use to preclinical studies, they validate the concept of dual targeting of the mutant EGFR receptor with allosteric and ATP-site inhibitors^22,23^. We have previously shown allosteric inhibitors to not be effective at targeting exon 19 deletion or kinase domain duplication variants^22,23^. However, JBJ-125 and JBJ-063 are effective as a single agent against genetically-engineered mouse and patient-derived xenograft models of L858R/T790M/C797S mutant EGFR. In combination with osimertinib, the prevent emergence of resistance in vitro^22,23^. These prior cellular and in vivo studies have focused on combinations with osimertinib, which is currently the front-line therapy for many EGFR-driven NSCLCs. Here, we identify the 3rd-generation TKIs mavelertinib and naquotinib as also being capable of cooperative binding and synergistic inhibition, demonstrating that this mechanism is not limited to osimertinib. However, since mavelertinib and naquotinib did not complete clinical trials as monotherapies, their potential as combination therapies with an allosteric EGFR inhibitor is unknown.

The dramatic synergy we characterize here suggests a further potential benefit to therapy with osimertinib in combination with a cooperative allosteric inhibitor—the ability to reduce the necessary dose of both agents and thereby mitigate toxicity due to their respective off-target activities. An important caveat in this respect is that the dose-limiting toxicity of osimertinib and other ATP-competitive EGFR TKIs is their activity on WT EGFR^40^, and we observe synergy with WT as well as mutant EGFR. Both osimertinib and JBJ-125 are mutant-selective; an elegant kinetic study of the mutant-selectivity of osimertinib revealed that it has a 45-fold wild-type selectivity margin for L858R/T790M and a 60-fold margin for L858R^41^. We suspect that the greater synergy we observe with mutant EGFR as compared with WT will further widen these selectivity margins, but it will be important to directly assess the mutant-selectivity of combinations with osimertinib and allosteric inhibitors prior to clinical application. The structural and mechanistic insights provided here will help guide development of a clinical-grade allosteric inhibitor for use as a single agent or in combination with osimertinib or other 3rd-generation EGFR TKIs.

## Methods

### Protein expression and purification

The human EGFR kinase domain, spanning residues 696–1022, was previously cloned into pTriEx with an N-terminal 6xHis-glutathione S-transferase (GST) fusion tag followed by a Tobacco etch virus (TEV) protease cleavage site. EGFR(L858R/T790M/F723A) was cloned into pFastBac with a TEV protease-cleavable, N-terminal 6xHis tag. Mutations were introduced via mismatch PCR and the resulting sequence verified via Sanger sequencing. The forward primer for F723A mutation was 5′-GTGCTGGGCTCCGGTGCG**GCC**GGCACGGTGTATAAGG-3′ and the reverse primer was 5′-CCTTATACACCGTGCC**GGC**CGCACCGGAGCCCAGCAC-3′. Recombinant baculovirus was prepared and used to infect Sf9 insect cells, which were harvested after 68–72 h. Cells were pelleted and resuspended in lysis buffer composed of 50 mM Tris pH 8.0, 500 mM NaCl, 1 mM tris(2-carboxyethyl)phosphine (TCEP), and 5% glycerol. Cells were lysed via sonication prior to ultracentrifugation at >200,000 *g* for 1 h. Imidazole pH 8.0 was added to the supernatant for a final concentration of 40 mM and flowed through a column containing Ni-NTA agarose beads. The resin was washed with lysis buffer supplemented with 40 mM imidazole and eluted with lysis buffer containing 200 mM imidazole. Eluted EGFR kinase domain was dialyzed overnight in the presence of 5% (w/w) TEV protease against dialysis buffer containing 50 mM Tris pH 8.0, 500 mM NaCl, 1 mM TCEP, and 5% glycerol. The cleaved protein was passed through Ni-NTA resin to remove the 6xHis-GST fusion protein and TEV protease prior to size exclusion chromatography on a prep-grade Superdex S200 (Cytiva) column in 50 mM Tris pH 8.0, 500 mM NaCl, 1 mM TCEP, and 5% glycerol. Fractions containing EGFR kinase of ≥95% purity as assessed by Coomassie-stained SDS-PAGE were concentrated to approximately 4 mg/mL as determined by Bradford assay.

### Crystallization and structure determination

For EGFR(T790M/V948R) crystal structures containing JBJ-125 or JBJ-063, purified kinase domain at approximately 3 mg/mL was incubated with 10 mM MgCl_2_, 1 mM adenylyl-imidodiphosphate (AMP-PNP), and 0.5 mM allosteric inhibitor (from a 10 mM DMSO stock) prior to crystallization via hanging drop vapor diffusion. Drops of 1 µL protein were combined with 1.5 µL of well solution comprised of 0.1 M Bis–Tris pH 5.7 and 20–30% (w/v) PEG 3350. Clusters of plate-like crystals grew within 2–3 days at room temperature. Crystals were moved to 2 µL drops of 0.1 M Bis–Tris pH 5.7 and 35% PEG 3350 over wells of the same composition. Prior to moving crystals, new drops were supplemented with 1 mM TCEP and 0.5 mM covalent, ATP-competitive inhibitor. Crystals were soaked overnight, briefly cryoprotected in a solution of 0.1 M Bis-Tris pH 5.7, 35% (w/v) PEG 3350, and 20% ethylene glycol, and flash frozen in liquid nitrogen.

EGFR(T790M/V948R) crystal structures containing DDC4002 or EAI045 were prepared by first growing AMP-PNP crystals as described above, omitting the allosteric inhibitor during hanging drop setup, and subsequently soaking the allosteric inhibitor at 1 mM into the crystals at the same time as the covalent ATP-site inhibitor. EGFR(L858R/V948R) crystals with JBJ-063 were grown under identical conditions by microseeding AMP-PNP EGFR(T790M/V948R) crystals while setting up vapor diffusion drops. Small crystals with a rough morphology grew in 5–7 days. These L858R/V948R crystals were crushed using a seed bead and microseeded into new L858R/V948R drops to obtain higher quality crystals. These higher quality crystals were used for soaking of osimertinib. EGFR(L858R/V948R) crystals were cryoprotected as described for T790M/V948R crystals.

Diffraction data were collected at 100 K at the Advanced Photon Source at the Argonne National Laboratory on NE-CAT beamlines 24-ID-C and 24-ID-E. Data were indexed, integrated, and scaled using either XDS or Dials via xia2 compiled through SBGrid^39–43^. Structures were phased via molecular replacement with PDB 5D41^20^. Crystals were typically in P2_1_ or C2 space groups but often became P1 after soaking and/or suffered from severe translational non-crystallographic symmetry. Refinement was performed using Phenix with iterative rounds of manual model building in Coot^44,45^. Ligand restraints were generated using eLBOW in Phenix using the AM1 quantum mechanical optimization method^46–49^. Structures have been deposited in the Protein Data Bank (PDB) with the accession codes 6XL4, 7JXI, 7JXK, 7JXL, 7JXM, 7JXP, 7JXW, 7K1I, 7K1H, and 7LG8 (Supplementary Table 1).

### Fluorescence polarization binding assay

Fluorescence polarization (FP) was used to assess binding of allosteric inhibitors in the presence of TKIs using a BODIPY-labeled allosteric inhibitor (BODIPY-JBJ, compound JBJ-09-052). Purified EGFR L858R and L858R/T790M kinase at 1 or 2 µM, respectively, was incubated with 10 equivalents of covalent inhibitor and 2 mM TCEP for 30 min at room temperature. After incubation, 10 nM BODIPY-JBJ was added to the protein and the protein serially diluted in buffer containing a 50 mM Tris pH 8.0, 500 mM NaCl, 2 mM TCEP, and 10 nM BODIPY-JBJ probe. FP was measured using a PHERAstar FS plate reader (BMG LABTECH) with an excitation wavelength of 485 nm and emission at 520 nm measured from 10 µL sample in black 384-well microplates. FP measurements were recorded every 2 min until equilibrium was reached, as indicated by stabilized signal reading.

### Kinase inhibition and synergy assays

Inhibition assays were performed using the HTRF KinEASE tyrosine kinase assay kit (Cisbio) according to the manufacturer’s protocol. Inhibitors (10 mM DMSO stocks) were dispensed into black 384-well plates using an HP D300e dispenser (Hewlett-Packard) and normalized to a 1% final DMSO concentration. For single inhibitor experiments, assay buffer containing purified EGFR at a final concentration of 5 nM (wild-type EGFR), 0.1 nM (L858R, L858R/C797S, and L858R/T790M/F723A), or 0.02 nM (L858R/T790M and L858R/T790M/C797S) was dispensed using a Multidrop Combi dispenser (ThermoFisher) and incubated at room temperature for 30 min. For inhibition synergy experiments with two inhibitors, double the aforementioned enzyme concentrations were used. Reactions were initiated with 100 µM ATP and allowed to proceed for 30 min at room temperature before being quenched using the detection reagent from the HTRF KinEASE assay kit. The FRET signal ratio was measured at 665 and 620 nm using a PHERAstar microplate reader (BMG LABTECH). Data were processed using GraphPad Prism and fit to a three-parameter dose-response model with a Hill slope constrained to −1. For synergy experiments, curves were manually inspected and inhibitor combinations without enough signal to produce a reliable dose-response curve were excluded.

### Inhibitor labeling time course

Purified EGFR(L858R) kinase domain at 0.2 mg/mL was preincubated on ice with 1 mM AMP-PNP and either 30-fold molar excess JBJ-125 or DMSO. Covalent TKI was added 1:1 to protein on ice and aliquots were quenched/denatured in 8 M urea + 1% formic acid (FA) at various time points. Samples were desalted over C4 resin prior to LCMS analysis. Denatured proteins (1 µg per time point) were analyzed by LC-MS via a U3000 RSLC coupled to an Orbitrap Eclipse (Thermo Fischer) mass spectrometer. Proteins were eluted off a ES811A 15-cm C4 column with a 5–50% gradient of MeCN in 1% FA. MS scans (range = 600–2000 *m/z*) were obtained in the orbitrap at 120,000 resolution with 4 microscans. The Xtract function in Freestyle software (Thermo Scientific, Waltham, MA, USA) was used to deconvolute charge states averaged across the elution window. Labeling experiments were performed twice.

### Cell lines and reagents

Ba/F3 cells stably expressing human EGFR mutant (L858R/T790M) were previously generated from parental Ba/F3 cells that were a generous gift from Dr. Weinstock’s Laboratory and were extensively characterized^14,20,22^ and cultured in RPMI1640 media supplemented with 10% fetal bovine serum (FBS) and 1% penicillin and streptomycin (P/S). H3255GR cells used for signaling experiments were derived from H3255 parental cells, which were previously purchased from ATCC, and were characterized extensively^35,50^ and maintained in modified ACL4 media (R&D Biosystems) supplemented with 5% FBS and 1% P/S. HEK293T/17 cells used for transient expression and subsequent pulldown studies were also purchased from ATCC and cultured in Dulbecco’s Modified Eagle’s medium (DMEM) in 10% FBS and 1% P/S. All cell lines were tested negative for *Mycoplasma* using the Mycoplasma Plus PCR Primer Set (Agilent) and were passaged and used for no longer than a month for all experiments.

### Plasmid construction

The L858R/T790M/F723A (L858R/T790M/F723A) EGFR mutant plasmid was generated by introducing the F723A mutation into the pDNR-dual EGFR (L858R/T790M) amplification vector that was generated previously in our laboratory using the QuikChange IIXL Site Mutagenesis Kit (Agilent). The forward mutagenesis primer was 5′-ATACACCGTGCCGGCCGCACCGGAGC-3′ and the reverse primer 5′-GGGCTCCGGTGCGGCCGGCACGGTGT-3′ The EGFR construct with the mutation was then shuttled into the JP1540 retroviral expression vector using the In-Fusion HD Cloning Plus kit (Takara).

### Pulldown assays using biotinylated compounds

Ba/F3 cells were plated and treated for 2 h with inhibitors indicated in the Fig. legends before cell lysis with NP40 lysis buffer and protein quantification by BCA assay. Lysates were incubated with 1 μM of biotinylated compounds (and corresponding biotinylated control) for 2 h before the addition of 50% Neutravidin beads for 1 h. Lysates containing the beads was then washed 3 times with PBS containing 1% IGEPAL (Sigma) before it was resuspended in 2x sample prep buffer and processed for Western Blotting.

### Transient transfection and western blotting

For transient overexpression and subsequent pulldown studies, HEK293T/17 cells were transfected with 2 µg of indicated plasmids using FuGENE HD Transfection Reagent from Promega in OPTI-MEM media, as per the manufacturer’s protocol. Media was changed after 24 h and cells were treated 48 h post-transfection with inhibitors for 2 h before they were harvested with NP-40 lysis buffer and used for pulldown studies. For downstream signaling assays, H3255GR were plated and treated for time and with inhibitors indicated in Fig. legends. Cells were harvested and lysed in RIPA lysis buffer followed by BCA protein assay to quantitate and normalize protein levels. Lysates were then processed for Western Blotting analyses. The EGFR antibody from Cell Signaling Technology (#54359) was used to examine the amount of EGFR that was pulled down with the biotinylated compounds and to detect EGFR expression level. To assess EGFR activity and its downstream signaling, we used phospho-EGFR (Tyr1068; #3777), phospho-Akt (Ser473; #4060L), Akt (#9272L), phospho-ERK1/2 (Thr202/Tyr204; #8544), ERK1/2 (#4695S) and Bim (#2933) antibodies from Cell Signaling Technology. Tubulin (Sigma; #T5168-.5ML) and HSP90 (Santa Cruz Technology; #S7947) were used as loading controls. All antibodies were used for Western Blotting at 1:1000 except for tubulin, which was used at 1:10,000.

Pulldown experiments with purified EGFR kinase domain were carried out in buffer composed of 50 mM Tris pH 8.0, 500 mM NaCl, 5% glycerol, and 1 mM TCEP. Protein samples were preincubated for 30 min with 5-fold excess TKI prior to the addition of 2-fold excess b-JBJ-125 and incubation for 30 min. Strep resin was added to each protein sample and rocked for 30 min to bind. The resin was washed three times with buffer containing 1% IGEPAL and the resin boiled in SDS gel loading dye before separation by SDS-PAGE and Coomassie staining.

### Reporting summary

Further information on research design is available in the Nature Research Reporting Summary linked to this article.

## Supplementary information

Supplementary Information

Peer Review File

Reporting Summary

## Data Availability

Source data are provided with this paper. All crystal structures are publicly available from the Protein Data Bank via the accession codes 6XL4 (osimertinib and DDC4002), 7JXI (mavelertinib), 7JXK (JBJ-125 and mavelertinib), 7JXL(AZ5104), 7JXM (osimertinib and EAI045), 7JXP (osimertinib and JBJ-125), 7JXW (osimertinib and JBJ-063), 7K1I (JBJ-063), 7K1H (osimertinib and JBJ-063), 7LG8 (naquotinib and JBJ-063), 4ZAU, 5D41, 6DUK, 3IKA, and 5FEQ.

## Source data

Source Data
