# Supplementary material for: Molecular basis for cooperative binding and synergy of ATP-site and allosteric EGFR inhibitors
Source: Nat Commun. 2022 May 9;13:2530. doi: 10.1038/s41467-022-30258-y (PMC9085736; doi:10.1038/s41467-022-30258-y)
Supplement: Supplementary file 1 — Supplementary Information [file 41467_2022_30258_MOESM1_ESM.pdf]

Supplementary Information

Molecular basis for cooperative binding and synergy between ATP-site and allosteric EGFR inhibitors

Tyler S. Beyett[1,2], Ciric To[3,4,5], David E. Heppner[1,2,6], Jaimin K. Rana[1,2], Anna M. Schmoker[1,2], Jaebong Jang[1,2,7], Dries J. H. De Clercq[1,2,8], Gabriel Gomez[1], David A. Scott[1,2], Nathanael S. Gray[1,2,9]*, Pasi A. Jänne[3,4,5]*, and Michael J. Eck[1,2]*

Affiliations:

[1] Department of Cancer Biology, Dana-Farber Cancer Institute, Boston, MA 02215, USA.

[2] Department of Biological Chemistry and Molecular Pharmacology, Harvard Medical School, Boston, MA 02115, USA.

[3] Lowe Center for Thoracic Oncology, Dana-Farber Cancer Institute, Boston, MA 02215, USA.

[4] Department of Medical Oncology, Dana-Farber Cancer Institute, Boston, MA 02215, USA.

[5] Department of Medicine, Harvard Medical School, Boston, MA 02115, USA.

[6] Current Address: Department of Chemistry, University of Buffalo, Buffalo, NY 14260, USA.

[7] Current Address: College of Pharmacy, Korea University, Korea.

[8] Current Address: CISTIM, Leuven, Belgium.

[9] Current Address: Department of Chemical and Systems Biology, CHeM-H, Stanford Cancer Institute, Stanford University, Stanford, CA 94305, USA.

These authors contributed equally to this work: Tyler S Beyett, Ciric To

*Corresponding Authors:
Michael J. Eck, MD, PhD
Email: michael_eck@dfci.harvard.edu

Pasi A. Jänne, MD, PhD
Email: pasi_janne@dfci.harvard.edu

Nathanael S. Gray, PhD
Email: nsgray01@stanford.edu

	TM/VR AZ5104	LR/VR JBJ-09-063	LR/VR Osimertinib JBJ-09-063	TM/VR Osimertinib EAI045	TM/VR Osimertinib JBJ-09-063	TM/VR Osimertinib JBJ-04-125-02	TM/VR Mavelertinib	TM/VR Mavelertinib JBJ-04-125-02	TM/VR Naquotinib JBJ-09-063	TM/VR Osimertinib DDC4002
PDB Accession	7JXL	7K1I	7K1H	7JXM	7JXW	7JXP	7JXI	7JXK	7LG8	6XL4
**Data Collection**										
Resolution range*	60.05 - 2.40 (2.49 - 2.40)	53.94 - 3.20 (3.32 - 3.20)	45.35 - 2.60 (2.695 - 2.60)	44.93 - 2.19 (2.27 - 2.19)	57.73 - 2.50 (2.59 - 2.50)	89.11 - 2.16 (2.24 - 2.16)	69.29 - 3.00 (3.11 - 3.00)	74.52 - 3.10 (3.21 - 3.10)	50.18 - 2.93 (3.03 - 2.93)	84.84 - 2.06 (2.13 - 2.06)
Space group	$P\ 1\ 2_1\ 1$	C 1 2 1	P 1	$P\ 1\ 2_1\ 1$	C 1 2 1	P 1	$P\ 1\ 2_1\ 1$	P 1	$P\ 1\ 2_1\ 1$	$P\ 1\ 2_1\ 1$
Cell dimensions										
a, b, c (Å)	71.0 101.3 87.0	148.0 35.8 54.8	66.8 88.3 93.8	72.55 103.2 87.0	170.2 73.1 119.1	57.1 94.7 95.6	70.6 100.8 87.2	66.9 87.4 91.3	55.7 75.9 151.0	71.5 101.7 86.9
α, β, γ (°)	90 101.4 90	90 100.2 90	118.2 87.0 106.7	90 101.5 90	90 118.1 90	70.6 78.4 79.2	90 101.2 90	62.1 85.6 73.3	90 94.4 90	90 102.7 90
Total reflections	329953 (33189)	31390 (2890)	197446 (19805)	216428 (17177)	301312 (30183)	295266 (14390)	154032 (13142)	106138 (10560)	101965 (9197)	521396 (52782)
Unique reflections	47145 (4716)	4805 (472)	52219 (5193)	62881 (5738)	44231 (4402)	81966 (4564)	23766 (2319)	28894 (2933)	26898 (2575)	74516 (7358)
Multiplicity	7.0 (7.0)	6.5 (6.1)	3.8 (3.8)	3.4 (3.0)	6.8 (6.9)	3.6 (3.2)	6.5 (5.7)	3.7 (3.6)	3.8 (3.6)	7.0 (7.2)
Completeness (%)	99.56 (99.70)	98.52 (96.92)	94.41 (93.67)	97.50 (89.44)	97.65 (97.70)	83.12 (46.01)	98.00 (93.04)	90.81 (91.06)	98.26 (94.45)	99.02 (98.41)
$I / \sigma I$	11.24 (1.83)	7.22 (1.50)	5.97 (1.01)	8.95 (1.35)	4.49 (0.65)	6.15 (1.19)	5.50 (0.97)	8.00 (1.23)	4.23 (0.65)	5.31 (1.24)
Wilson B-factor	43.14	114.32	55.84	42.36	46.28	39.09	59.67	67.09	61.99	30.95
R-merge	0.172 (1.442)	0.140 (0.870)	0.155 (1.155)	0.208 (0.875)	0.271 (1.737)	0.110 (1.105)	0.242 (1.223)	0.157 (0.937)	0.228 (1.203)	0.204 (1.726)
R-pim	0.070 (0.584)	0.060 (0.372)	0.093 (0.682)	0.132 (0.600)	0.112 (0.705)	0.066 (0.711)	0.102 (0.552)	0.094 (0.565)	0.133 (0.725)	0.082 (0.687)
CC1/2	0.996 (0.761)	0.993 (0.729)	0.992 (0.642)	0.973 (0.626)	0.993 (0.83)	0.994 (0.581)	0.989 (0.649)	0.994 (0.645)	0.979 (0.57)	0.993 (0.764)
**Refinement**										
Reflections used in refinement	47125 (4714)	4799 (472)	52179 (5192)	62863 (5735)	43655 (4330)	81750 (4533)	23645 (2231)	28748 (2892)	26760 (2534)	74325 (7325)
Reflections used for R-free	2307 (250)	259 (22)	1993 (199)	1992 (183)	2080 (191)	4040 (214)	1175 (117)	1454 (166)	1344 (140)	3627 (357)
R-work	0.212 (0.271)	0.255 (0.402)	0.232 (0.363)	0.231 (0.328)	0.275 (0.352)	0.214 (0.325)	0.277 (0.357)	0.222 (0.297)	0.235 (0.331)	0.215 (0.321)
R-free	0.240 (0.329)	0.307 (0.352)	0.270 (0.403)	0.270 (0.378)	0.318 (0.367)	0.247 (0.360)	0.313 (0.397)	0.262 (0.351)	0.270 (0.340)	0.252 (0.375)
Number of non-hydrogen atoms	9668	2400	14644	9753	9737	15137	9311	14507	9222	9977
macromolecules	9405	2328	14082	9359	9368	14636	9191	14323	8885	9526
ligands	144	72	462	187	308	202	120	184	324	178
solvent	119	0	100	207	61	299	0	0	13	273
Protein residues	1163	288	1743	1159	1159	1783	1136	1748	1100	1193
RMS(bonds)	0.009	0.003	0.005	0.014	0.01	0.009	0.003	0.004	0.003	0.005
RMS(angles)	1.16	0.63	0.97	1.22	1.2	1.15	0.77	0.79	0.74	0.86
Ramachandran favored (%)	96.49	96.81	96.45	97.1	96.14	97.1	96.76	96.69	95.91	95.97
Ramachandran allowed (%)	3.16	2.84	3.2	2.64	3.6	2.78	3.24	2.96	3.81	3.26
Ramachandran outliers (%)	0.35	0.35	0.35	0.26	0.26	0.11	0	0.35	0.28	0.77
Rotamer outliers (%)	3.45	2.7	3.4	4.14	4.03	3.32	3.23	1.85	6.54	3.34
Average B-factor	51.1	148.35	65.19	51.85	70.88	56.24	59.5	76.75	61.25	40.71
macromolecules	51.18	148.91	65.45	51.98	71.48	56.68	59.64	76.64	61.32	40.74
ligands	50.63	130	58.84	51.19	57.1	46.71	49.31	85.34	59.93	38.72
solvent	45.49		58.2	46.76	48.8	47.23			44.81	40.72

*Values in parentheses are for highest-resolution shell.

Supplementary Table 1. Crystallographic data collection and refinement statistics.

Supplementary Figures

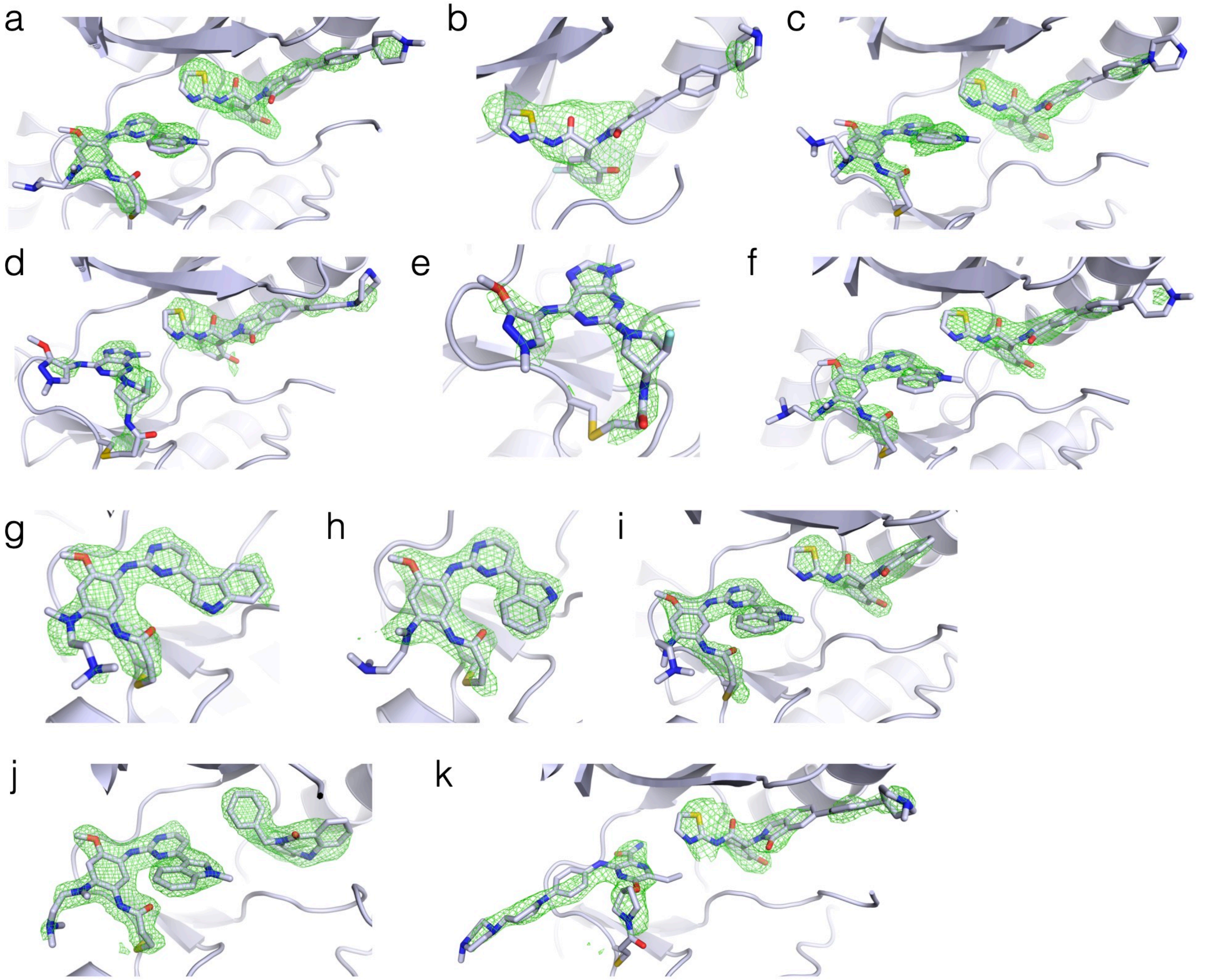

Supplementary Fig. 1. Inhibitor ligand densities for determined structures. **a** EGFR (L858R/V948R) in complex with osimertinib and JBJ-063 (PDB 7K1H). **b** EGFR (L858R/V948R) in complex with JBJ-063 (7K1I). **c** EGFR (T790M/V948R) in complex with osimertinib and JBJ-125 (PDB 7JXP). **d** EGFR (T790M/V948R) in complex with mavelertinib and JBJ-125 (PDB 7JXK). **e** EGFR (T790M/V948R) in complex with mavelertinib (7JXI). **f** EGFR (T790M/V948R) in complex with osimertinib and JBJ-063 (PDB 7JXW). **g** EGFR (T790M/V948R) in complex with AZ5104 in the flipped conformation (PDB 7JXL) and **h** canonical conformation. **i** EGFR (T790M/V948R) in complex with osimertinib and EAI045 (PDB 7JXM). **j** EGFR (T790M/V948R) in complex with osimertinib and DDC4002 (PDB 6XL4). **k** EGFR (T790M/V948R) in complex with naquotinib and JBJ-063 (PDB 7LG8). All are positive F_o-F_c omit maps (green) contoured at 3 sigma, with the exception of B which is at 2.5 sigma. The phosphate-binding loop (P-loop) has been hidden for clarity.

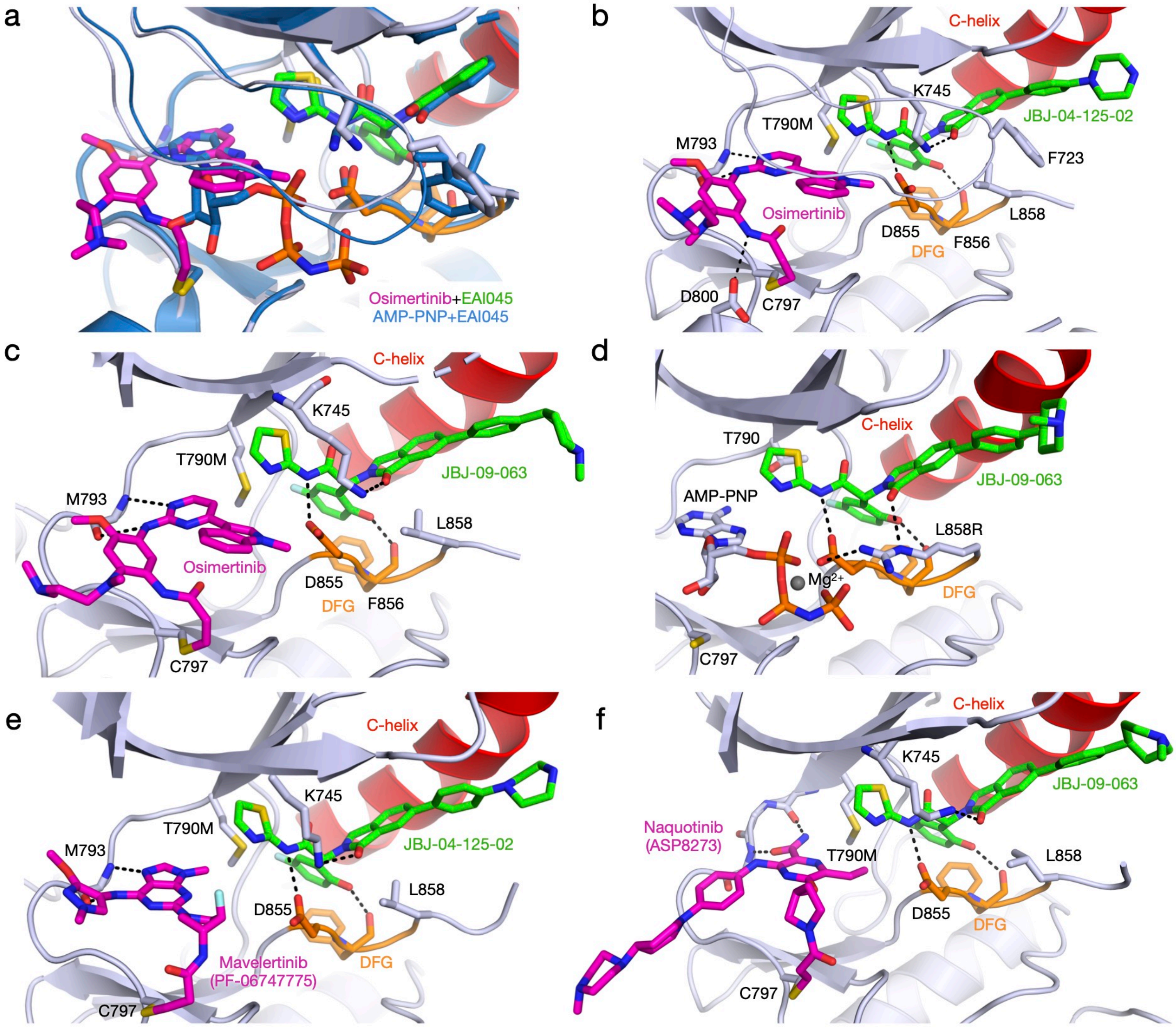

Supplementary Fig. 2. Crystal structures of EGFR kinase in complex with inhibitors. **a** Comparison between EGFR(T790M/V948R) in complex with AMP-PNP+EAI045 (blue, PDB 6P1L) and osimertinib+EAI045. **b** Crystal structure of EGFR(T790M/V948R) with osimertinib and JBJ-125. **c** Crystal structure of EGFR(T790M/V948R) with osimertinib and JBJ-063. **d** Crystal structure of EGFR(L858R/V948R) with osimertinib and JBJ-063. **e** Mavelertinib and JBJ-125 in EGFR(T790M/V948R). **f** Naquotinib and JBJ-063 in EGFR(T790M/V948R). The C-helix is colored red, Asp-Phe-Gly (DFG) motif in orange, ATP-site inhibitor in magenta, and allosteric inhibitor in green.

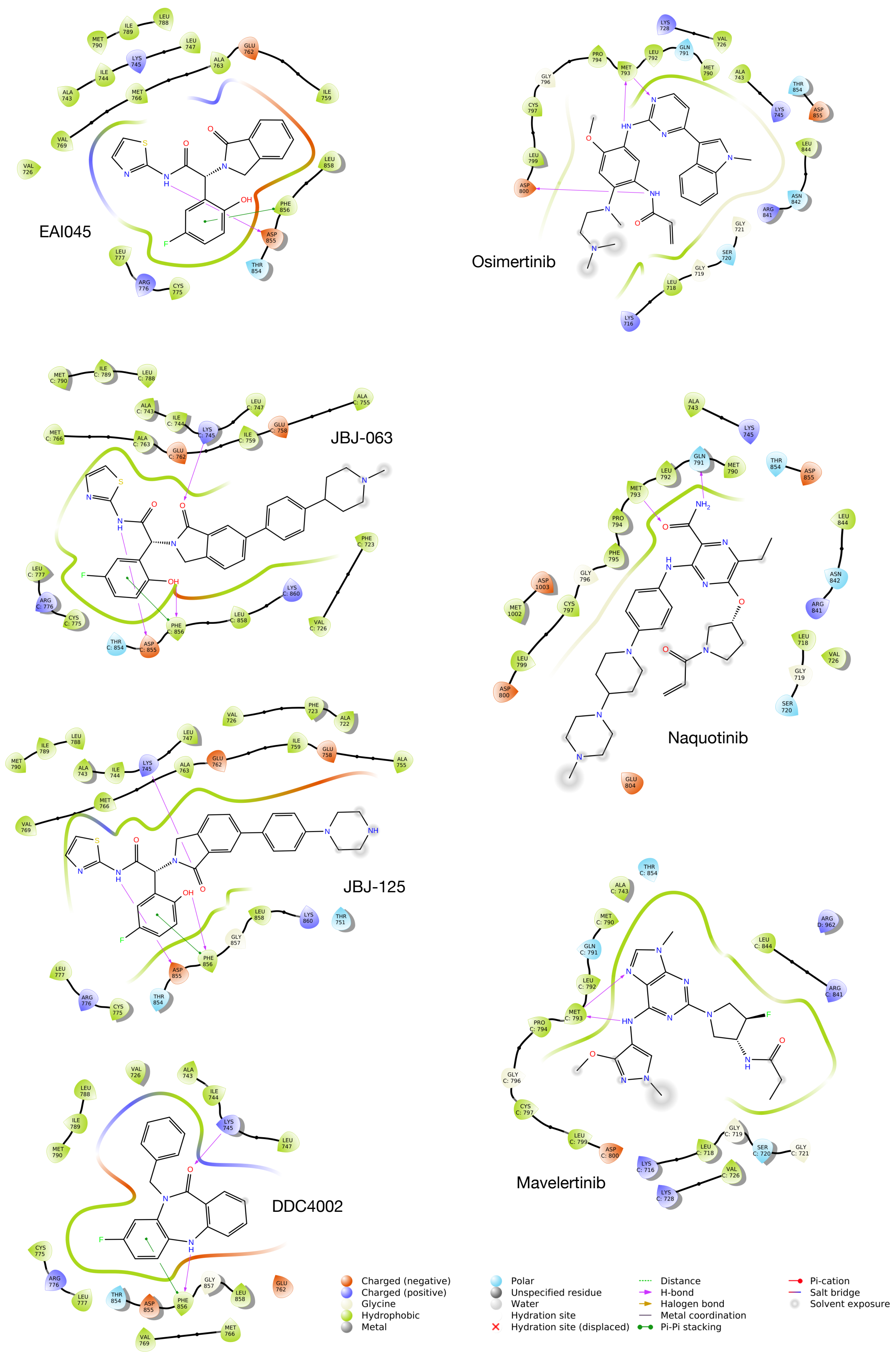

Supplementary Fig. 3. Representative interaction diagrams for the ligands found in crystal structures. Figures were generated via Maestro (Schrödinger Inc.).

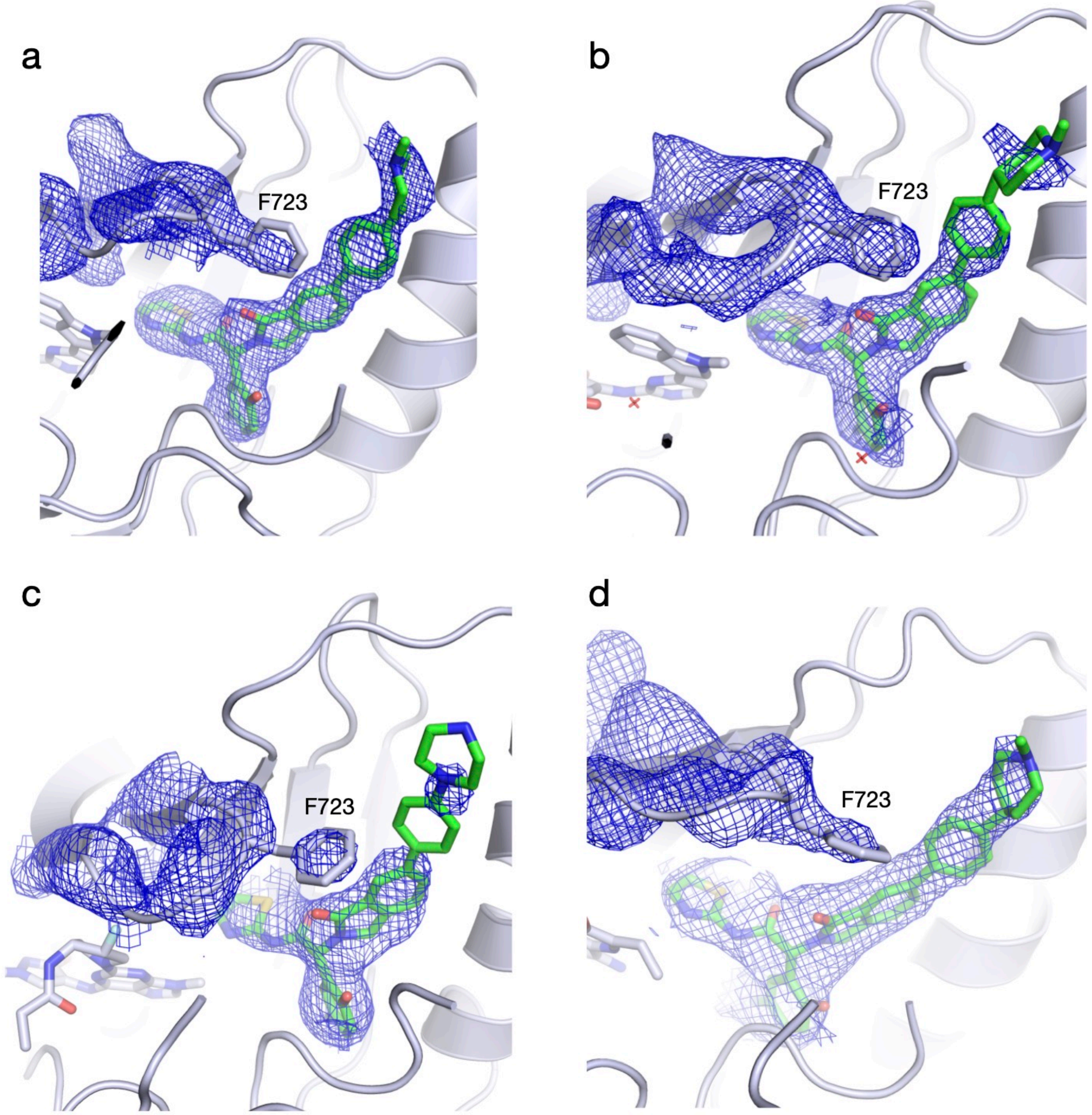

Supplementary Fig. 4. Composite omit maps of the P-loop region and allosteric inhibitor in structures containing two inhibitors. **a** EGFR(LR/VR) in complex with osimertinib and JBJ-063. **b** EGFR(TM/VR) in complex with osimertinib and JBJ-063. **c** EGFR(TM/VR) in complex with mavelertinib and JBJ-125. **d** EGFR(TM/VR) in complex with naquotinib and JBJ-063. Maps are $2F_o$-F_c at 1 sigma.

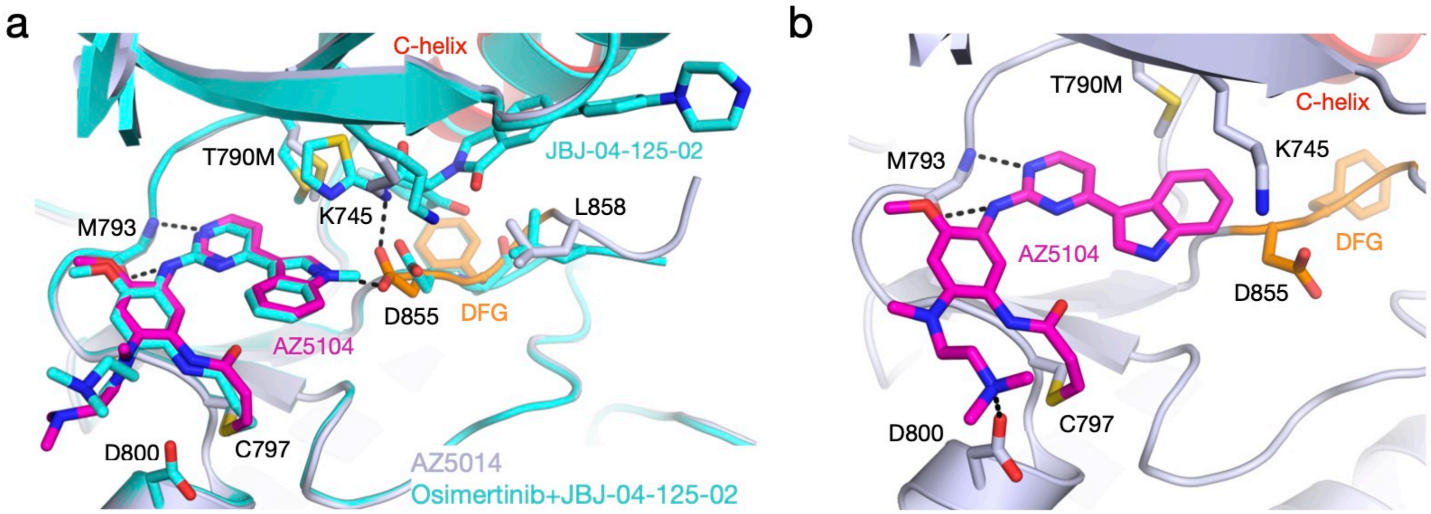

Supplementary Fig. 5. Crystal structure with AZ5104. **a** Overlay of EGFR (T790M/V948R) in complex with AZ5104 (gray, magenta, and orange PDB 7JXL) or osimertinib and JBJ-125 (cyan, PDB 7JXP). **b** Chain A of the AZ5104 crystal structure with an unusual flipped indole ligand conformation.

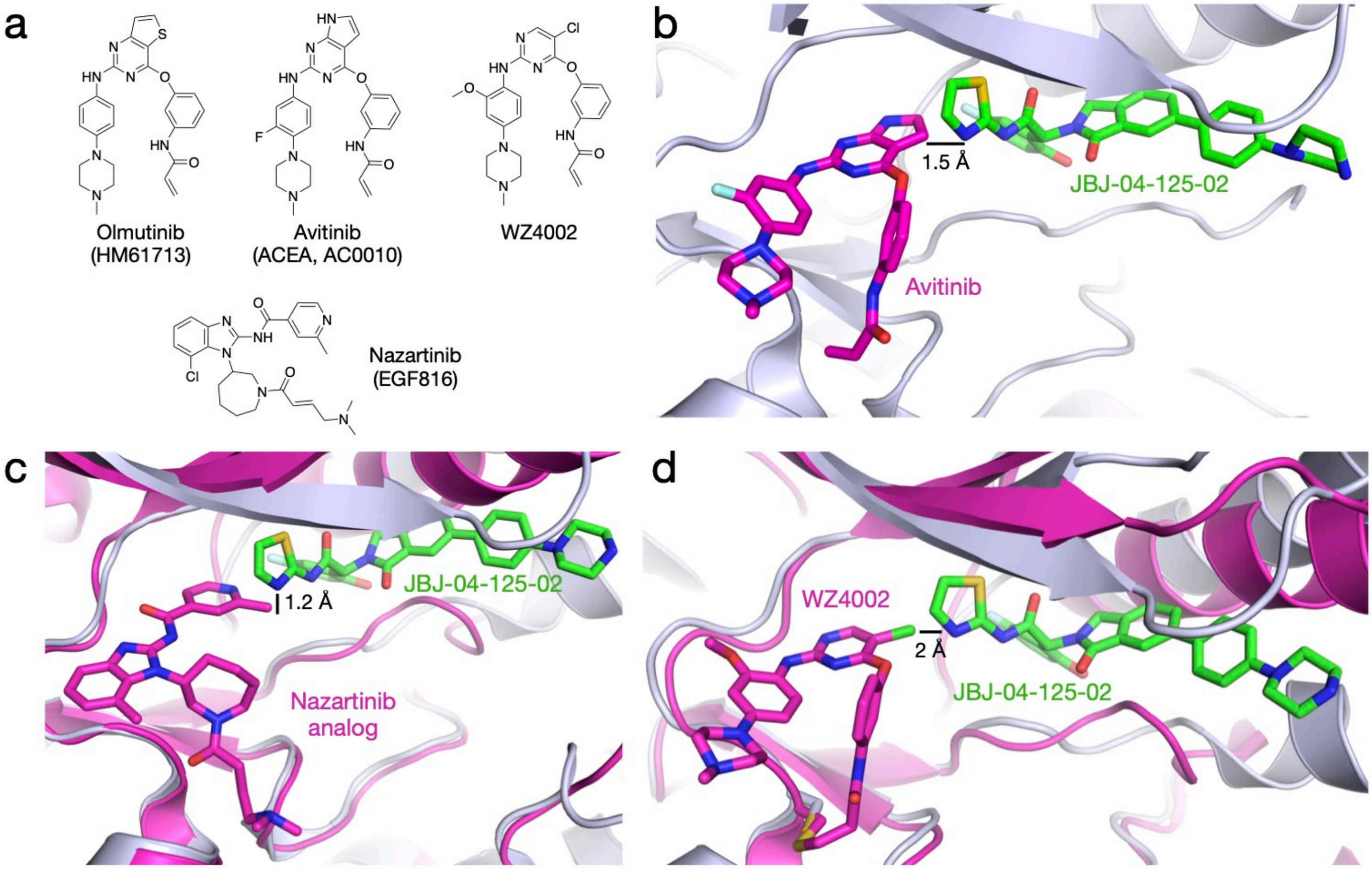

Supplementary Fig. 6. Steric clashes between select TKIs and allosteric inhibitors. **a** Chemical structures of TKIs that do not co-bind with the allosteric inhibitor. **b** Avitinib modeled into an EGFR structure in complex with JBJ-125 (PDB 6DUK). The steric clash between inhibitors is noted with a distance. **c** Overlay of EGFR in complex with a close analog of nazartinib (magenta, PDB 5FEQ) and EGFR in complex with JBJ-125 (gray). **d** EGFR in complex with WZ4002 (magenta, PDB 3IKA) aligned with EGFR in complex with JBJ-125 (gray).

a

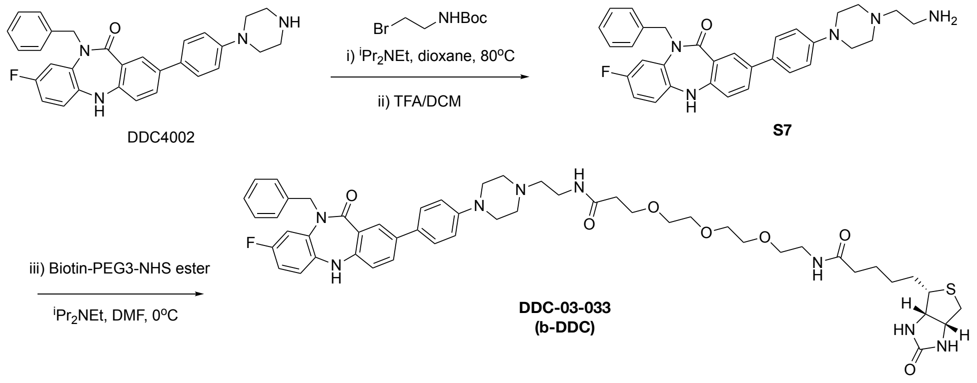

b

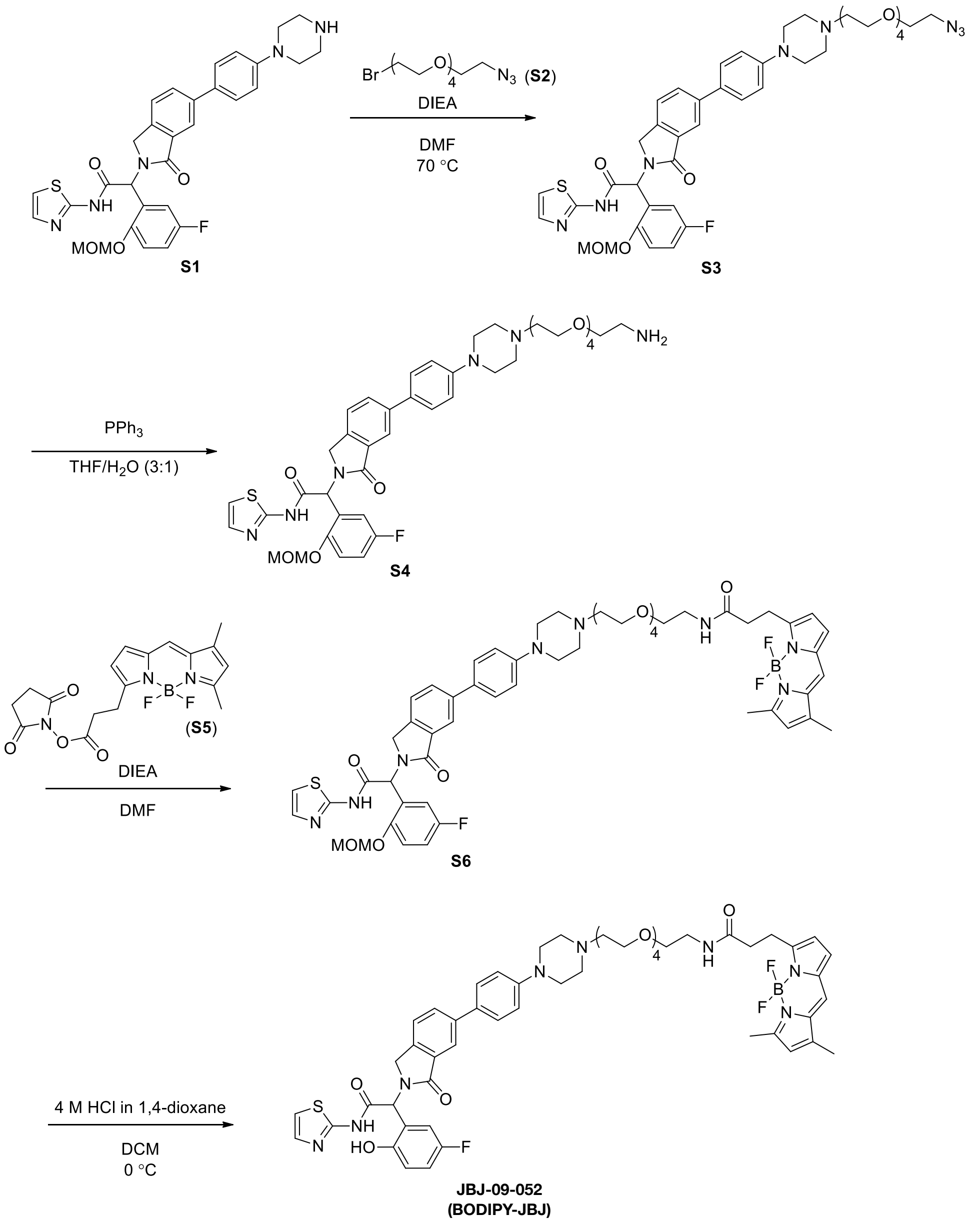

Supplementary Fig. 7. Synthetic schemes for chemical probes. **a** Synthetic scheme for biotinylated dibenzodiazepinone inhibitor DDC-03-033 (b-DDC). **b** Synthetic scheme for BODIYP-labeled JBJ-125 (BODIPY-JBJ, JBJ-09-052).

a

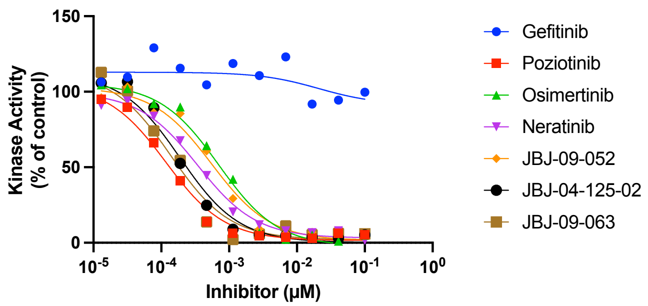

	L858R/T790M/ F723A	L858R/T790M
Gefitinib	>100	>100
Poziotinib	0.12	0.08
Osimertinib	0.70	0.39
Neratinib	0.34	0.10
JBJ-09-052	0.55	0.98
JBJ-04-125-02	0.19	0.16
JBJ-09-063	0.14	0.13

b

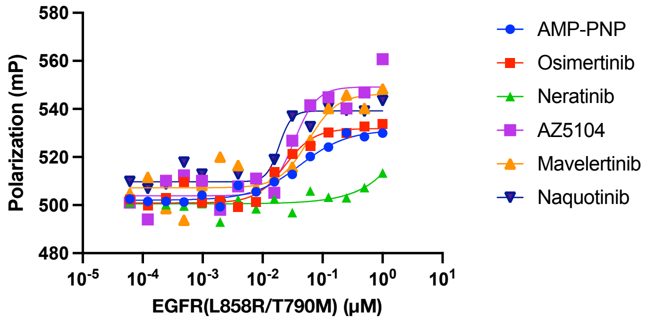

Supplementary Fig. 8. Biochemical and biophysical evaluation of inhibitors. **a** Representative dose-response curves for EGFR(L858R/T790M) and potencies (nM) for both L858R/T790M and L858R/T790M/F723A variants. Data include the FP probe JBJ-09-052 (BODIPY-JBJ). Potencies were determined using an HTRF assay and fit to a three-parameter dose-response curve model in Prism (average, n=1 independent experiment with 3 technical replicates). **b** Representative FP binding curves with purified EGFR(L858R/T790M). Kinase was incubated with nucleotide or covalent TKI prior to the addition of BODIPY-JBJ (n=1 independent experiment shown). Source data are provided as a Source Data file.

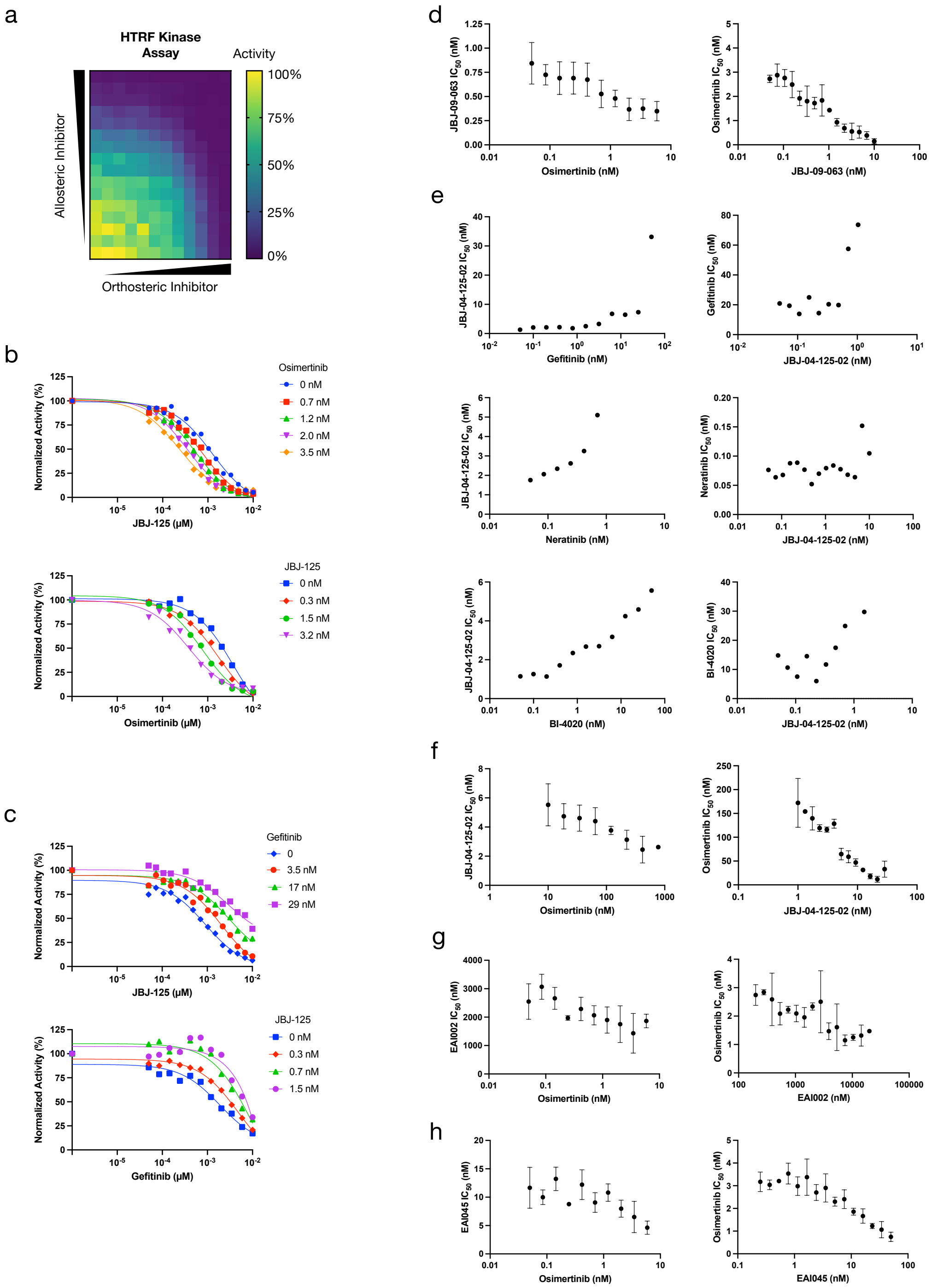

Supplementary Fig. 9. Inhibitor synergy experiments. **a** Example heat map illustrating assay setup. **b** Representative dose-response curves for a synergistic combination (titration of one inhibitor at varying constant concentrations of the other). Data were normalized to the maximum signal with only one inhibitor present to better show the potency shift due to the loss of total signal from a constant concentration of a second inhibitor (n=1 independent experiment). **c**, Representative dose-response curves for an antagonistic combination (gefitinib and JBJ-125) (n=1 independent experiment). **d** Inhibition synergy between osimertinib and JBJ-09-063 (JBJ-063) in EGFR(L858R) (mean ± SD, n=3 independent experiments). **e** Antagonism between JBJ-04-125-02 (JBJ-125) and gefitinib or neratinib in EGFR(L858R) or BI-4020 in EGFR(L858R/C797S) (mean, n=2 independent experiments). **f** Synergy between osimertinib and JBJ-125 in WT EGFR (mean ± SD, n=3 independent experiments). **g** Synergy analysis between EAI002 and JBJ-125 in EGFR(L858R) (mean ± SD, n=3 independent experiments). **h**, Synergy analysis between EAI045 and JBJ-125 in EGFR(L858R) (mean ± SD, n=3 independent experiments). Source data are provided as a Source Data file.

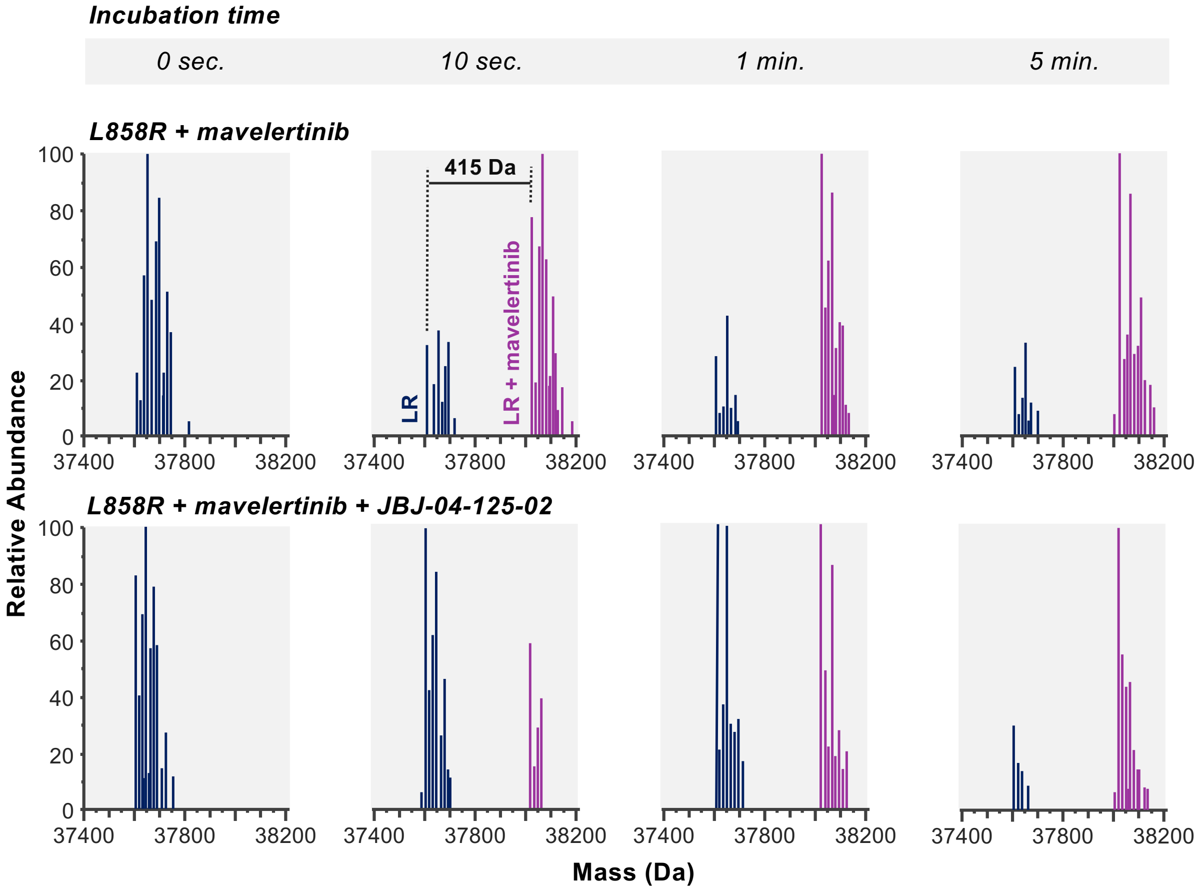

Supplementary Fig. 10. Representative intact LC/MS results for labeling of EGFR(L858R) over time with mavelertinib in the presence or absence of JBJ-125. Representative total ion counts obtained from deconvolved charge states are plotted at 0 s, 10 s, 1 min, and 5 min incubations times (n=2 independent experiments). Unlabeled EGFR(L858R) is dark blue and inhibitor-labeled EGFR(L858R) is dark purple. Source data are provided as a Source Data file.

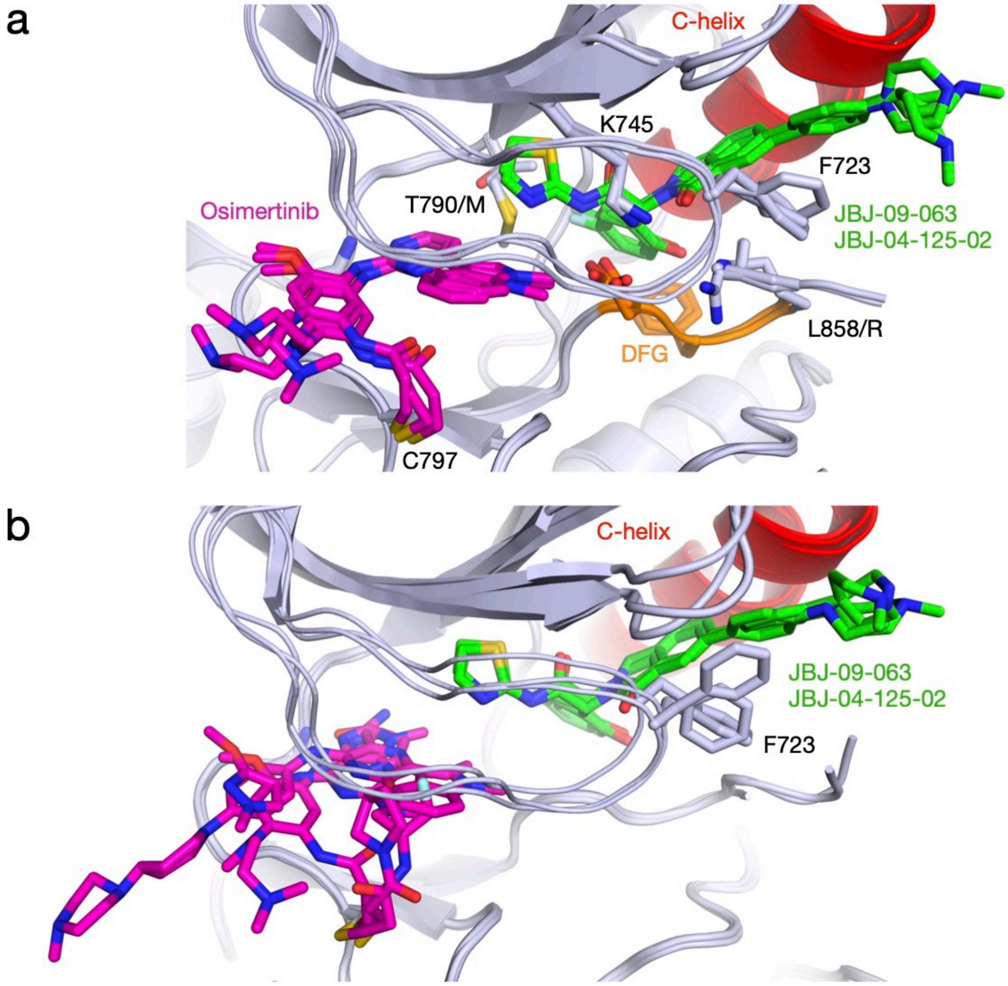

Supplementary Fig. 11. Overlay of inhibitor determined combination structures. **a** L858R/V948R and T790M/V948R EGFR structures with osimertinib and JBJ-125 or JBJ-063. **b** Crystal structures with osimertinib, mavelertinib, or naquotinib in combination with JBJ-125 or JBJ-063. The C-helix is colored red, DFG motif in orange, ATP-site inhibitor in magenta, and allosteric inhibitor in green.

a

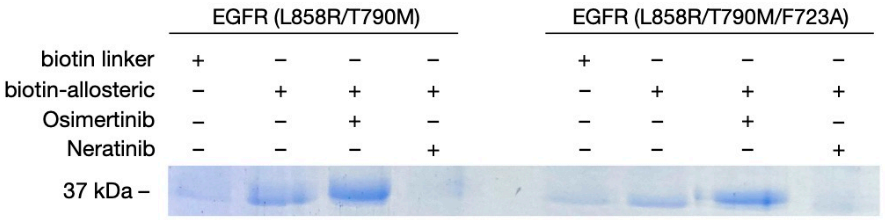

b

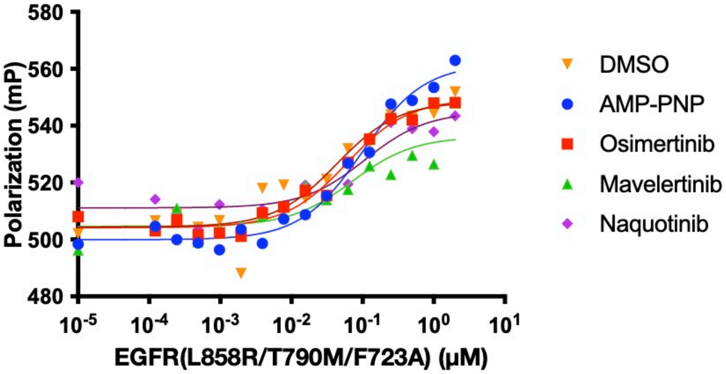

Supplementary Fig. 12. Biochemical studies of the F723A variant. **a** Pulldown of purified EGFR kinase domain with b-JBJ-125 (biotin-allosteric). F723A attenuated pulldown of protein treated with osimertinib (n=2 independent experiments). **b** Representative FP binding curves of BODIPY-JBJ to purified EGFR(L858R/T790M/F723A) (mean, n=2 independent experiments). Source data are provided as a Source Data file.

Supplementary Methods

Synthesis of chemical probes:

EAI045, DDC4002, JBJ-04-125-02 (JBJ-125), and JBJ-09-063 (JBJ-063) were synthesized for previous studies[1-4]. Other TKIs were purchased from commercial vendors and are ≥95% pure per their analyses. Starting materials, reagents and solvents were purchased from commercial suppliers and were used without further purification unless otherwise noted. All reactions were monitored using a Waters Acquity UPLC/MS system (Waters PDA eλ Detector, QDa Detector, Sample manager - FL, Binary Solvent Manager) using Acquity UPLC® BEH C18 column (2.1 x 50 mm, 1.7 µm particle size): solvent gradient = 85 % A at 0 min, 1 % A at 1.7 min; solvent A = 0.1 % formic acid in Water; solvent B = 0.1 % formic acid in Acetonitrile; flow rate : 0.6 mL/min. Reaction products were purified by flash column chromatography using CombiFlash®Rf with Teledyne Isco Redi*Sep*® normal-phase silica flash columns (4 g, 12 g, 24 g, 40 g or 80 g) and Waters HPLC system using SunFire™ Prep C18 column (19 x 100 mm, 5 µm particle size): solvent gradient = 80 % A at 0 min, 10 % A at 25 min; solvent A = 0.035 % TFA in Water; solvent B = 0.035 % TFA in MeOH; flow rate: 25 mL/min. ^{1}H NMR spectra were recorded on 500 MHz Bruker Avance III spectrometers.

The synthesis of BODIPY-JBJ (JBJ-09-052) was as follows (Supplemental Fig. 7b):
JBJ-04-125-02 (**S1**) was synthesized for a previous study but can be purchased from commercial vendors[3].

Synthesis of 2-(6-(4-(4-(14-azido-3,6,9,12-tetraoxatetradecyl)piperzin-1-yl)phenyl)-1-oxoisoindolin-2-yl)-2-(5-fluoro-2-(methoxymethoxy)phenyl)-*N*-(thiazol-2-yl)acetamide (**S3**). To a solution of **S1** (100 mg, 0.17 mmol) in DMF were added DIEA (118 µL, 0.68 mmol) and 1-azido-14-bromo-3,6,9,12-tetraoxatetradecane (**S2,** 56 mg, 0.17 mmol). After stirring for 6 hr, the reaction mixture was purified by prepHPLC to obtain the compound **S3** (88 mg, 62%).

Synthesis of 2-(6-(4-(4-(14-amino-3,6,9,12-tetraoxatetradecyl)piperazin-1-yl)phenyl)-1-oxoisoindolin-2-yl)-2-(5-fluoro-2-(methoxymethoxy)phenyl)-*N*-(thiazol-2-yl)acetamide (**S4**). Compound S3 (70 mg, 0.084 mmol) was dissolved in a mixture of THF (1 mL) and H_2O (0.3 mL) and triphenylphosphine (67 mg, 0.25 mmol) was added. After stirring for 6 hr, the reaction mixture was purified by prepHPLC to afford compound **S4** (58 mg, 85%).

Synthesis of 5,5-difluoro-7-(1-(4-(4-(2-(1-(5-fluoro-2-hydroxyphenyl)-2-oxo-2-(thiazol-2-ylamino)ethyl)-3-oxoisoindolin-5-yl)phenyl)piperazin-1-yl)-16-oxo-3,6,9,12-tetraoxa-15-azaoctadecan-18-yl)-1,3-dimethyl-5*H*-dipyrrolo[1,2-c:2',1'-f][1,3,2]diazaborinin-4-ium-5-uide (**JBJ-09-052, BODIPY-JBJ**). To a solution of **S4** (25 mg, 0.031 mmol) in DMF (0.5 mL) were added DIEA (18 µL, 0.10 mmol) and BODIPY NHS ester (**S5**, 10 mg, 0.026 mmol). After stirring for 3 hr, the reaction mixture was diluted with EtOAc and washed with brine and water five times. The organic layer was dried over Na_2SO_4, filtered and concentrated under reduced pressure.
The crude residue was dissolved in DCM (0.5 mL) and 4 M HCl in 1,4-dioxane (0.5 mL) was added to the reaction mixture. After stirring for 30 min, the resulting mixture was purified by prepHPLC to obtain **JBJ-09-052** (9.6 mg, 30%). ^{1}H NMR (500 MHz, DMSO-d_6) δ 12.61 (s, 1H), 10.95 (s, 1H), 10.09 (s, 1H), 8.04 (t, *J* = 5.6 Hz, 1H), 7.88 (s, 1H), 7.85 (dd, *J* = 8.0, 1.6 Hz, 1H), 7.68 (s, 1H), 7.65 (d, *J* = 8.8 Hz, 2H), 7.60 (d, *J* = 8.0 Hz, 1H), 7.48 (d, *J* = 3.6 Hz, 1H), 7.27 (d, *J* = 3.5 Hz, 1H), 7.10 (d, *J* = 9.0 Hz, 2H), 7.13 – 7.07 (m, 1H), 6.98 (dd, *J* = 8.9, 4.6 Hz, 1H), 6.85 (dd, *J* = 9.2, 3.1 Hz, 1H), 6.35 (d, *J* = 4.1 Hz, 1H), 6.33 (s, 1H), 6.29 (s, 1H), 4.62 (d, *J* = 17.5 Hz, 1H), 4.00 (d, *J* = 17.6 Hz, 1H), 3.92 – 3.84 (m, 4H), 3.63 – 3.34 (m, 16H), 3.27 – 3.18 (m, 6H), 3.07 (t, *J* = 7.7 Hz, 2H), 2.52 – 2.47 (m, 4H), 2.45 (s, 3H), 2.24 (s, 3H); LC/MS (ESI) m/z 1037.25 $[M+H]^+$.

Synthesis of N-(15-(4-(4-(10-benzyl-8-fluoro-11-oxo-10,11-dihydro-5H-dibenzo[b,e][1,4]diazepin-2-yl)phenyl)piperazin-1-yl)-12-oxo-3,6,9-trioxa-13-azapentadecyl)-5-((3aS,4S,6aR)-2-oxohexahydro-1H-thieno[3,4-d]imidazol-4-yl)pentanamide (**b-DDC, DDC-03-033**, Supplemental Fig. 7a) began with addition of Boc-protected ethylamine to DDC4002[2] followed by deprotection to yield **S7**. Biotin-PEG3-NHS ester was reacted with **S7** to yield **b-DDC** (**DDC-03-033**): ^{1}H NMR (500 MHz, DMSO-d_6) δ 7.94 (s, 1H), 7.84 – 7.81 (m, 3H), 7.63 (dd, *J* = 8.2, 2.1 Hz, 1H), 7.47 (d, *J* = 8.3 Hz, 2H), 7.34 – 7.28 (m, 4H), 7.26 (dd, *J* = 10.3, 2.7 Hz, 1H), 7.21 (m, 1H), 7.14 – 7.11 (m, 2H), 7.00 (d, *J* = 7.1 Hz, 2H), 6.91 (m, 1H), 6.41 (br s, 1H), 6.35 (br s, 1H), 5.30 (s, 2H), 4.30 (dd, *J* = 7.5, 5.2 Hz, 1H), 4.13 – 4.11 (m, 1H), 3.61 (t, *J* = 6.4 Hz, 2H), 3.52 – 3.48 (m, 8H), 3.39 (t, *J* = 6.0 Hz, 2H), 3.24 – 3.15 (m, 7H), 3.11 – 3.07 (m, 1H), 2.81 (dd, *J* = 12.2, 4.8 Hz, 1H), 2.59 – 2.52 (m, 4H), 2.40 (m, 2H), 2.33 (m, 2H), 2.06 (t, *J* = 7.6 Hz, 2H), 1.64 – 1.58 (m, 1H), 1.53 – 1.44 (m, 3H), 1.34 – 1.27 (m, 2H); LC/MS (ESI) m/z 951.32 $[M+H]^+$.

Supplementary References

1. Jia, Y. et al. Overcoming EGFR(T790M) and EGFR(C797S) resistance with mutant-selective allosteric inhibitors. Nature **534**, 129–132 (2016).
2. de Clercq, D. J. H. et al. Discovery and Optimization of Dibenzodiazepinones as Allosteric Mutant-Selective EGFR Inhibitors. ACS Medicinal Chemistry Letters **10**, 1549–1553 (2019).
3. To, C. et al. Single and Dual Targeting of Mutant EGFR with an Allosteric Inhibitor. Cancer Discovery **9**, 926–943 (2019).
4. To, C. et al. An allosteric inhibitor against the therapy-resistant mutant forms of EGFR in non-small cell lung cancer. Nature Cancer (2022).
