# Supplementary material for: Molecular basis for cooperative binding and synergy of ATP-site and allosteric EGFR inhibitors
Source: Nat Commun. 2022 May 9;13:2530. doi: 10.1038/s41467-022-30258-y (PMC9085736; doi:10.1038/s41467-022-30258-y)
Supplement: Supplementary file 3 — Reporting Summary [file 41467_2022_30258_MOESM3_ESM.pdf]

nature research

Corresponding author(s): Michael J Eck, Pasi A Janne, Nathanael S Gray

Last updated by author(s): April 2, 2022

# Reporting Summary

Nature Research wishes to improve the reproducibility of the work that we publish. This form provides structure for consistency and transparency in reporting. For further information on Nature Research policies, see our Editorial Policies and the Editorial Policy Checklist.

## Statistics

For all statistical analyses, confirm that the following items are present in the figure legend, table legend, main text, or Methods section.

| n/a | Confirmed | |
|---|---|---|
| ☐ | ☒ | The exact sample size ($n$) for each experimental group/condition, given as a discrete number and unit of measurement |
| ☐ | ☒ | A statement on whether measurements were taken from distinct samples or whether the same sample was measured repeatedly |
| ☒ | ☐ | The statistical test(s) used AND whether they are one- or two-sided<br>*Only common tests should be described solely by name; describe more complex techniques in the Methods section.* |
| ☐ | ☒ | A description of all covariates tested |
| ☒ | ☐ | A description of any assumptions or corrections, such as tests of normality and adjustment for multiple comparisons |
| ☐ | ☒ | A full description of the statistical parameters including central tendency (e.g. means) or other basic estimates (e.g. regression coefficient) AND variation (e.g. standard deviation) or associated estimates of uncertainty (e.g. confidence intervals) |
| ☒ | ☐ | For null hypothesis testing, the test statistic (e.g. $F$, $t$, $r$) with confidence intervals, effect sizes, degrees of freedom and $P$ value noted<br>*Give P values as exact values whenever suitable.* |
| ☒ | ☐ | For Bayesian analysis, information on the choice of priors and Markov chain Monte Carlo settings |
| ☒ | ☐ | For hierarchical and complex designs, identification of the appropriate level for tests and full reporting of outcomes |
| ☒ | ☐ | Estimates of effect sizes (e.g. Cohen's $d$, Pearson's $r$), indicating how they were calculated |

*Our web collection on statistics for biologists contains articles on many of the points above.*

## Software and code

Policy information about availability of computer code

| | |
|---|---|
| Data collection | No software was used to collect data. |
| Data analysis | Dose response and FB binding data analyses were done using GraphPad Prism 9. Crystallography data were indexed, integrated, and scaled using Dials (v.1.11.2) via xia2 (v.0.5.653). Refinement was performed using Phenix (v.1.14 3260) with iterative rounds of manual model building in Coot (v.0.9.6). Ligand restraints were generate via eLBOW in Phenix (v.1.14 3260). FreeStyle (v1.8) was used to analyze mass spectrometry data. |

For manuscripts utilizing custom algorithms or software that are central to the research but not yet described in published literature, software must be made available to editors and reviewers. We strongly encourage code deposition in a community repository (e.g. GitHub). See the Nature Research guidelines for submitting code & software for further information.

## Data

Policy information about availability of data

All manuscripts must include a data availability statement. This statement should provide the following information, where applicable:

- Accession codes, unique identifiers, or web links for publicly available datasets
- A list of figures that have associated raw data
- A description of any restrictions on data availability

Source data are provided with this paper. All crystal structures are publicly available from the Protein Data Bank via the accession codes: 6XL4 [http://doi.org/10.2210/pdb6XL4/pdb] (osimertinib and DDC4002), 7JXI [http://doi.org/10.2210/pdb7JXI/pdb] (mavelertinib), 7JXK [http://doi.org/10.2210/pdb7JXK/pdb] (JBJ-125 and mavelertinib), 7JXL [http://doi.org/10.2210/pdb7JXL/pdb] (AZ5104), 7JXM [http://doi.org/10.2210/pdb7JXM/pdb] (osimertinib and EAI045), 7JXP [http://doi.org/10.2210/pdb7JXP/pdb] (osimertinib and JBJ-125), 7JXW [http://doi.org/10.2210/pdb7JXW/pdb] (osimertinib and JBJ-063), 7K1I [http://doi.org/10.2210/pdb7K1I/pdb] (JBJ-063), 7K1H [http://doi.org/10.2210/pdb7K1H/pdb] (osimertinib and JBJ-063), 7LG8 [http://doi.org/10.2210/pdb7LG8/pdb]

(naquotinib and JBJ-063), 4ZAU [http://doi.org/10.2210/pdb4ZAU/pdb], 5D41 [http://doi.org/10.2210/pdb5D41/pdb], 6DUK [http://doi.org/10.2210/pdb6DUK/pdb], 3IKA [http://doi.org/10.2210/pdb3IKA/pdb], and 5FEQ [http://doi.org/10.2210/pdb5FEQ/pdb].

# Field-specific reporting

Please select the one below that is the best fit for your research. If you are not sure, read the appropriate sections before making your selection.

- [x] Life sciences
- [ ] Behavioural & social sciences
- [ ] Ecological, evolutionary & environmental sciences

For a reference copy of the document with all sections, see nature.com/documents/nr-reporting-summary-flat.pdf

# Life sciences study design

All studies must disclose on these points even when the disclosure is negative.

**Sample size**

Three independent experiments were performed for all cellular assays and at least two experiments were performed for synergy and fluorescence polarization biochemical assays. These are standard sample sizes for similar published experiments (To et al. Cancer Discovery, 2019; Jia et al. Nature, 2016). Inhibitor dose-response assays were performed once with technical replicates. This is similar to prior publications on allosteric inhibitors (To et al. Nature Cancer 2022, De Clercq et al. ACS Medchem Lett, 2019).

**Data exclusions**

As noted in the Inhibition Synergy methods section, drug combinations not producing sufficient signal for the accurate fitting of a dose-response function were excluded based on manual inspection. No other data were excluded from any experiment.

**Replication**

All attempts at replication were successful. Biochemical assays were performed n=2 or n=3 (biological replicates) with technical replicates for inhibition and fluorescence polarization assays. This is consistent with prior published work on allosteric inhibitors (Jia, et al. Nature, 2016). All cellular experiments were repeated 3 times (biological replicates).

**Randomization**

Samples were not randomly placed in treatment or control groups. The uniformity of the cell lines and purified proteins used does not warrant randomization to minimize bias between groups. This decision was also made on the precedence set by other studies on allosteric inhibitors utilizing the same cell lines and enzyme assays (To et al. Cancer Discovery, 2019; Jia et al. Nature, 2016; To et al. Nature Cancer 2022).

**Blinding**

Blinding is not utilized in similar biochemical and cellular experiments on EGFR inhibitors. Drug treatment and vehicle controls were performed via an automated drug dispenser negating the need for blinding since all samples were handled the same way by the machine without human intervention.

# Reporting for specific materials, systems and methods

We require information from authors about some types of materials, experimental systems and methods used in many studies. Here, indicate whether each material, system or method listed is relevant to your study. If you are not sure if a list item applies to your research, read the appropriate section before selecting a response.

## Materials & experimental systems

| n/a | Involved in the study |
|---|---|
| [ ] | [x] Antibodies |
| [ ] | [x] Eukaryotic cell lines |
| [x] | [ ] Palaeontology and archaeology |
| [x] | [ ] Animals and other organisms |
| [x] | [ ] Human research participants |
| [x] | [ ] Clinical data |
| [x] | [ ] Dual use research of concern |

## Methods

| n/a | Involved in the study |
|---|---|
| [x] | [ ] ChIP-seq |
| [x] | [ ] Flow cytometry |
| [x] | [ ] MRI-based neuroimaging |

## Antibodies

**Antibodies used**

Phospho-EGFR (Tyr1068; #3777), EGFR (#54359), phospho-Akt (Ser473; #4060L), Akt (#9272L), phospho-ERK1/2 (Thr202/Tyr204; #8544), ERK1/2 (#4695S), Bim (#2933) were purchased from Cell Signaling Technology. The tubulin (T5168-.5ML) and HSP90 (S7947) antibodies were purchased from Sigma and Santa Cruz Biotechnology respectively. All antibodies were used for Western Blotting. All antibodies were used at 1:1000 except for tubulin, which was used at 1:10,000.

**Validation**

Each primary antibody was validated to cross react with the species from which the cell lines and tumors were derived. Detailed validation data, relevant citation and pertinent information regarding the antibody are provided on the manufacturer's websites.

# Eukaryotic cell lines

Policy information about cell lines

| | |
|---|---|
| Cell line source(s) | Mutant EGFR Ba/F3 cells are from mouse and were derived from parental Ba/F3 cells that was a generous gift from Dr. Weinstock's Laboratory. Hek293T/Cl.17 and H3255GR cells, which were derived from H3255 parental cells, are from human and were previously purchased from ATCC. |
| Authentication | All cell lines are not authenticated at this time. |
| Mycoplasma contamination | All cell lines were tested negative for Mycoplasma using Mycoplasma Plus PCR Primer Set (Agilent] and were passaged and/or used for no longer than 4 weeks for all experiments. |
| Commonly misidentified lines (See ICLAC register) | No commonly misidentified lines were used in our studies. |
